# Genome-wide association meta-analysis of 30,000 samples identifies seven novel loci for quantitative ECG traits

**DOI:** 10.1038/s41431-018-0295-z

**Published:** 2019-01-24

**Authors:** Jessica van Setten, Niek Verweij, Hamdi Mbarek, Maartje N. Niemeijer, Stella Trompet, Dan E. Arking, Jennifer A. Brody, Ilaria Gandin, Niels Grarup, Leanne M. Hall, Daiane Hemerich, Leo-Pekka Lyytikäinen, Hao Mei, Martina Müller-Nurasyid, Bram P. Prins, Antonietta Robino, Albert V. Smith, Helen R. Warren, Folkert W. Asselbergs, Dorret I. Boomsma, Mark J. Caulfield, Mark Eijgelsheim, Ian Ford, Torben Hansen, Tamara B. Harris, Susan R. Heckbert, Jouke-Jan Hottenga, Annamaria Iorio, Jan A. Kors, Allan Linneberg, Peter W. MacFarlane, Thomas Meitinger, Christopher P. Nelson, Olli T. Raitakari, Claudia T. Silva Aldana, Gianfranco Sinagra, Moritz Sinner, Elsayed Z. Soliman, Monika Stoll, Andre Uitterlinden, Cornelia M. van Duijn, Melanie Waldenberger, Alvaro Alonso, Paolo Gasparini, Vilmundur Gudnason, Yalda Jamshidi, Stefan Kääb, Jørgen K. Kanters, Terho Lehtimäki, Patricia B. Munroe, Annette Peters, Nilesh J. Samani, Nona Sotoodehnia, Sheila Ulivi, James G. Wilson, Eco J. C. de Geus, J. Wouter Jukema, Bruno Stricker, Pim van der Harst, Paul I. W. de Bakker, Aaron Isaacs

**Affiliations:** 1 Division Heart & Lungs, Department of Cardiology, University Medical Center Utrecht, University of Utrecht, Utrecht, The Netherlands; 2 Department of Cardiology, University Medical Center Groningen, University of Groningen, Groningen, The Netherlands; 3 Program in Medical and Population Genetics, Broad Institute of MIT and Harvard, Cambridge, MA USA; 4 Department of Biological Psychology, Amsterdam Public Health Research Institute, Vrije Universiteit Amsterdam, Amsterdam, The Netherlands; 5 Department of Epidemiology, Erasmus MC, Rotterdam, The Netherlands; 6 Department of Internal Medicine, Section of Gerontology and Geriatrics, Leiden University Medical Center, Leiden, The Netherlands; 7 McKusick-Nathans Institute of Genetic Medicine, Johns Hopkins University School of Medicine, Baltimore, MD USA; 8 Cardiovascular Health Research Unit, Department of Medicine, University of Washington, Seattle, WA USA; 9 Research Unit, AREA Science Park, Trieste, Italy; 10 The Novo Nordisk Foundation Center for Basic Metabolic Research, Faculty of Health and Medical Sciences, University of Copenhagen, Copenhagen, Denmark; 11 Department of Cardiovascular Sciences, University of Leicester, Leicester, England; 12 NIHR Leicester Biomedical Research Centre, Glenfield Hospital, Groby Road, Leicester, UK; 13 CAPES Foundation, Ministry of Education of Brazil, Brasília, DF 70040-020 Brazil; 14 Department of Clinical Chemistry, Fimlab Laboratories, and Finnish Cardiovascular Research Center - Tampere, Faculty of Medicine and Life Sciences, University of Tampere, 33520 Tampere, Finland; 15 Center of Biostatistics and Bioinformatics, University of Mississippi Medical Center, Jackson, MS 39216 USA; 16 Institute of Genetic Epidemiology, Helmholtz Zentrum München - German Research Center for Environmental Health, Neuherberg, Germany; 17 Department of Medicine I, Ludwig-Maximilians-Universität, Munich, Germany; 18 DZHK (German Centre for Cardiovascular Research), Partner Site Munich Heart Alliance, Munich, Germany; 19 Chair of Genetic Epidemiology, IBE, Faculty of Medicine, LMU Munich, Germany; 20 Human Genetics Research Centre, ICCS, St George’s University of London, London, UK; 21 Institute for Maternal and Child Health, IRCCS “Burlo Garofolo”, Trieste, Italy; 22 Icelandic Heart Association, Kopavogur, Iceland; 23 Faculty of Medicine, University of Iceland, Reykavik, Iceland; 24 William Harvey Research Institute, Barts and The London School of Medicine & Dentistry, Queen Mary University of London, London, UK; 25 NIHR Barts Cardiovascular Research Centre, Barts and The London School of Medicine & Dentistry, Queen Mary University of London, London, UK; 26 Durrer Center for Cardiovascular Research, Netherlands Heart Institute, Utrecht, The Netherlands; 27 Institute of Cardiovascular Science, Faculty of Population Health Sciences, and Farr Institute of Health Informatics Research and Institute of Health Informatics, University College London, London, UK; 28 Department of Nephrology, University Medical Center Groningen, Groningen, The Netherlands; 29 Robertson Centre for Biostatistics, University of Glasgow, Glasgow, UK; 30 Laboratory of Epidemiology, Demography and Biometry, National Institute on Aging, Bethesda, MD USA; 31 Cardiovascular Health Research Unit and Department of Epidemiology, University of Washington, Seattle, WA USA; 32 Cardiovascular Department, “Ospedali Riuniti and University of Trieste”, Trieste, Italy; 33 Department of Medical Informatics, Erasmus University Medical Center, Rotterdam, The Netherlands; 34 Center for Clinical Research and Prevention, Bispebjerg and Frederiksberg Hospital-The Capital Region, Copenhagen, Denmark; 35 Department of Clinical Medicine, Faculty of Health and Medical Sciences, University of Copenhagen, Copenhagen, Denmark; 36 Institute of Health and Wellbeing, University of Glasgow, Glasgow, UK; 37 Institute of Human Genetics, Helmholtz Zentrum München - German Research Center for Environmental Health, Neuherberg, Germany; 38 Institute of Human Genetics, Technische Universität München, Munich, Germany; 39 Department of Clinical Physiology and Nuclear Medicine, Turku University Hospital, and Research Centre of Applied and Preventive Cardiovascular Medicine, University of Turku, Turku, 20520 Finland; 40 Genetic Epidemiology Unit, Department of Epidemiology, Erasmus MC, University Medical Center Rotterdam, Rotterdam, The Netherlands; 41 Doctoral Program in Biomedical Sciences, Universidad del Rosario, Bogotá, Colombia; 42 Institute of translational Medicine-IMT-Center For Research in Genetics and Genomics-CIGGUR, GENIUROS Research Group, School of Medicine and Health Sciences, Universidad del Rosario, Rosario, Colombia; 43 Epidemiological Cardiology Research Center (EPICARE), Department of Epidemiology and Prevention, Wake Forest University School of Medicine, Winston-Salem, NC USA; 44 CARIM School for Cardiovascular Diseases, Maastricht University, Maastricht, The Netherlands; 45 Maastricht Centre for Systems Biology (MaCSBio), Maastricht University, Maastricht, The Netherlands; 46 Department of Biochemistry, Maastricht University, Maastricht, The Netherlands; 47 Department of Internal Medicine, Erasmus University Medical Center, Rotterdam, The Netherlands; 48 Research unit of Molecular Epidemiology, Helmholtz Zentrum München - German Research Center for Environmental Health, Neuherberg, Germany; 49 Institute of Epidemiology II, Helmholtz Zentrum München - German Research Center for Environmental Health, Neuherberg, Germany; 50 Department of Epidemiology, Rollins School of Public Health, Emory University, Atlanta, GA USA; 51 DSM, University of Trieste, Trieste, Italy; 52 IRCCS-Burlo Garofolo Children Hospital, Via dell’Istria 65, Trieste, Italy; 53 Laboratory of Experimental Cardiology, University of Copenhagen, Copenhagen, Denmark; 54 German Center for Diabetes Research, Neuherberg, Germany; 55 Cardiovascular Health Research Unit, Division of Cardiology, University of Washington, Seattle, WA USA; 56 Department of Physiology and Biophysics, University of Mississippi Medical Center, Jackson, MS USA; 57 Department of Cardiology, Leiden University Medical Center, Leiden, The Netherlands; 58 Department of Genetics, University Medical Center Groningen, University of Groningen, Groningen, The Netherlands; 59 Durrer Center for Cardiogenetic Research, ICIN-Netherlands Heart Institute, Utrecht, The Netherlands; 60 Department of Genetics, University Medical Center Utrecht, Utrecht, The Netherlands; 61 Department of Epidemiology, Julius Center for Health Sciences and Primary Care, University Medical Center Utrecht, Utrecht, The Netherlands

**Keywords:** Genome-wide association studies, Quantitative trait

## Abstract

Genome-wide association studies (GWAS) of quantitative electrocardiographic (ECG) traits in large consortia have identified more than 130 loci associated with QT interval, QRS duration, PR interval, and heart rate (RR interval). In the current study, we meta-analyzed genome-wide association results from 30,000 mostly Dutch samples on four ECG traits: PR interval, QRS duration, QT interval, and RR interval. SNP genotype data was imputed using the Genome of the Netherlands reference panel encompassing 19 million SNPs, including millions of rare SNPs (minor allele frequency < 5%). In addition to many known loci, we identified seven novel locus-trait associations: *KCND3*, *NR3C1*, and *PLN* for PR interval, *KCNE1*, *SGIP1*, and *NFKB1* for QT interval, and *ATP2A2* for QRS duration, of which six were successfully replicated. At these seven loci, we performed conditional analyses and annotated significant SNPs (in exons and regulatory regions), demonstrating involvement of cardiac-related pathways and regulation of nearby genes.

## Introduction

Quantitative electrocardiographic (ECG) traits have been well studied in large consortia, identifying over 130 significant loci. Some loci were associated with multiple traits. Nevertheless, these loci collectively explain only a small portion of the genetic variation of these traits [1]. Large GWAS meta-analyses on PR interval [2, 3], RR interval/heart rate [4, 5], QRS duration [6, 7], and QT interval [8–10] were based on HapMap imputations [11]. Testing ~2.5 million SNPs, these studies provided good coverage of common variation in the genome. SNPs with lower allele frequencies (e.g., minor allele frequencies between 1 and 5%), however, are poorly covered [12, 13]. While HapMap included only 270 samples (30 trios and 90 unrelated samples) from three continental populations [11], the 1000 Genomes Project Phase 3 contains 2504 samples from 26 populations [14]. Larger reference panels cover a broader variety of haplotypes and, therefore, increase the quality of imputation in a GWAS sample. Moreover, the number of observed SNPs also increases, expanding the number available for imputation. This has led to novel findings in non-ECG related studies [15].

In the current study, we meta-analyzed genome-wide data on four ECG traits in 30,000 predominantly Dutch samples. We tested over 19 million SNPs for association, which were imputed using the Genome of the Netherlands (GoNL) reference panel [16]. This dataset contains whole-genome sequencing data at 12x coverage collected in 250 families (trios and parents with two offspring). Nearly all polymorphic sites with a population frequency of more than 0.5% are captured. This makes it one of the largest single population sequencing efforts worldwide and the trio design ensures very accurate haplotype phasing. These features and the good match with the predominantly Dutch cohorts, make this dataset well suited as a reference panel for imputation. Using this approach, we had two aims: (1) the discovery of novel loci associated with ECG traits, and (2) the fine-mapping and functional annotation of known regions associated with ECG traits. We increased our SNP density almost seven-fold compared to previous studies based on HapMap, enabling us to study key signals in much finer detail.

## Methods

### Individual cohort data

Eight cohorts were included in the discovery phase of this study, totaling approximately 30,000 samples (Supplementary Tables 1 and 2, Supplementary Notes). Most study participants were Dutch with the exception of most participants of PROSPER; this study included approximately 19% samples of Dutch origin, while the remaining samples were of other European descent. All cohorts performed stringent quality control to exclude low-quality samples and SNPs prior to imputation and also post-imputation. Imputation was performed using 998 phased haplotypes from the Genome of the Netherlands Project release 4 as the reference panel, encompassing 19,763,454 SNPs [16]. All genomic data in this manuscript is listed with respect to the hg19 (build37) reference genome.

We evaluated four phenotypes on the electrocardiogram: RR interval, PR interval, QRS duration, and QT interval. Seven out of eight cohorts contributed data to all four phenotypes; NTR only had data on RR interval available. Samples of non-European descent and samples with missing data were excluded, as well as individuals that fulfilled any of the exclusion criteria listed in Supplementary Table 3. SNPs were individually tested for association with each trait using linear models. For all four phenotypes, we included age, sex, height, BMI, and study specific covariates (for instance to correct for study site, relatedness, or population stratification) as covariates. In addition, RR interval and hypertension (in those cohorts that had data available on this measure) were included as covariates for QT interval to reduce noise introduced by these factors. We chose these covariates to correspond with previously published GWAS on these four ECG traits.

### Quality control and meta-analysis

Association results from all cohorts were collected at a single site and underwent quality control. SNPs with extreme values of beta (>1000 or <−1000), standard error (SE) (>1000), or imputation quality (<0.1 or >1.1) were removed and distributions of beta, SE, and *P*-values were manually checked. We made QQ-plots to test *P*-value distributions, which were stratified by minor allele frequency and by imputation quality. Aberrant subsets of SNPs (usually with very low frequency) were removed from downstream analyses.

Inverse-variance fixed-effect model meta-analyses were conducted for all four traits using MANTEL [17]. For each individual GWAS, genomic inflation factors (lambda) were calculated and, during meta-analysis, standard errors were adjusted accordingly to correct for population structure and technical errors. We did not correct for genomic inflation after meta-analysis. SNP associations were considered significant if *P* ≤ 5 × 10^–8^.

### Follow-up on known and novel loci

For each locus, we tested the number of independent signals using the LD structure from GoNL in GCTA-COJO, which was designed to allow conditional analyses based on summary-level data [18]. Secondary hits had to fulfill two criteria: (1) genome-wide significant in the GWAS, and (2) *P* < 1 × 10^–5^ after conditioning to correct for multiple testing of 4757 significant SNPs across all four traits. A novel locus for a trait was defined if the significant SNPs, or SNPs within a distance of 1 Mb upstream and downstream of the significant SNPs, had not been observed before in GWAS of the same trait. We performed a look-up of all novel loci in previous HapMap-based GWAS.

### Replication of novel loci in CHARGE

We sought to replicate our findings in 13 independent cohorts taking part in the CHARGE consortium [19] (Supplementary Tables 1 and 2, Supplementary notes). Twelve studies (TwinsUK, CHS, ARIC, KORA F3, KORA S4, JHS, AGES, BRIGHT, YFS, INGI-FVG, and INGI-CARL) used 1000 Genomes Phase 1 as their imputation reference panel and a single study (Inter99) provided only genotyped data. All studies contained samples of European ancestry, except for JHS, which consisted only of African-American samples. The summary-level results for all novel SNPs determined in the discovery analysis were combined in inverse-variance fixed-effects meta-analyses. A two-sided *P*-value ≤ 0.05, in conjunction with a concordant effect direction, was considered significant.

### In silico tests of possibly functional SNPs

We looked up the functional annotations for all SNPs that reached genome-wide significance in any of the four traits. First, we checked whether SNPs were potentially damaging to protein function, testing all non-synonymous SNPs in SIFT [20] and PolyPhen-2 [21]. Second, we used GREAT [22] to identify biological pathways in which regulatory SNPs are involved, testing the index SNPs for all locus-trait associations. Lastly, we tested all significant SNPs one by one for their possible effect on regulatory regions using RegulomeDB [23].

## Results

### Meta-analysis detects novel loci

We conducted a GWAS meta-analysis comprising eight cohorts that together encompassed approximately 30,000 samples. Over 19 million SNPs, imputed using the GoNL reference panel, were assessed for association with four quantitative ECG traits: RR, PR, QRS, and QT. Considering all traits, we observed 52 locus-phenotype associations (17 for PR, 13 for QRS, 15 for QT, and 7 for RR; Supplementary Figures 1 and 2, Supplementary Table 4). A locus was defined as an associated region (containing one or more SNPs with *P* ≤ 5 × 10^–8^) that is located at least 1 Mb away from the next (i.e., if two associated SNPs are within 1 Mb, they belong to the same locus). Of these 52 loci, 45 have been observed before in large GWAS meta-analyses [2–4, 7–9] and seven are novel findings (Table 1). Box 1 shows regional association plots and provides additional information on the seven novel loci. Imputation qualities of the index SNPs were 0.60 and 0.84 for the relatively rare *KCNE1* and *KCDN1* variants, respectively, and >0.96 for the remaining common index SNPs. The variance explained by each of these variants ranges between 0.09 and 0.23%.

**Table 1:** Meta-analyses in 30,000 samples identify seven novel loci for PR interval, QRS duration, and QT interval

SNP info					GoNL-imputed data							Previous HapMap-based meta-analysis				Replication in 13 CHARGE cohorts (1000 Genomes Phase 1 imputed)			
Locus	Trait	Index SNP	Chr	Position (hg19)	Coded allele	Non-coded allele	Coded allele frequency	Beta	SE	*P*-value	Sample size	Proxy used	*P*-value	Sample size	Refs.	Beta	SE	*P*-value	Sample size
KCND3	PR	rs75013985	1	112530430	G	A	0.033	−4.090	0.554	1.5 × 10^−13^	31695	No proxies available with *r*^2^ > 0.4	N/A	92340	[3]	−5.967	0.985	1.4 × 10^−9^	19,302
NR3C1/ARHGAP26	PR	rs17287745	5	142655015	G	A	0.425	1.011	0.185	4.2 × 10^−8^	31695	No	1.9 × 10^−6^	92340	[3]	0.585	0.193	0.002	24,438
PLN/SLC35F1	PR	rs74640693	6	118684824	T	A	0.049	2.376	0.428	2.9 × 10^−8^	31695	rs10457327 (*r*^2^ = 0.89)	2.9 × 10^−4^	92340	[3]	0.457	0.419	0.276	27,106
SGIP1	QT	rs6588213	1	67107894	T	C	0.126	1.596	0.282	1.5 × 10^−8^	26794	No	0.001	76061	[10]	0.757	0.199	1.4 × 10^−4^	22,663
NFKB1	QT	rs11097788	4	103407428	G	A	0.561	1.048	0.186	1.8 × 10^−8^	26794	rs1598856 (*r*^2^ = 0.97)	1.3 × 10^−4^	76061	[10]	0.336	0.131	0.010	30,504
KCNE1	QT	rs1805128	21	35821680	T	C	0.018	7.409	0.939	2.9 × 10^−15^	26794	No	0.004	76061	[10]	4.874	0.671	3.7 × 10^−13^	15,896
ATP2A2/ANAPC7	QRS	rs28637922	12	110819139	T	G	0.259	0.565	0.102	3.0 × 10^−8^	25509	rs1502337 (*r*^2^ = 0.89)	4.1 × 10^−4^	73518	[6]	0.177	0.074	0.027	29,427

Using GoNL as reference panel in ~30,000 samples mostly of Dutch descent, we found seven loci not previously identified or (in the case of *KCNE1* for QT interval) not consistently replicated in previous genome-wide association studies. We conducted look-ups of these SNPs (or proxy SNPs in strong LD if the SNPs were not present in HapMap) in their respective HapMap-based meta-analyses and replicated six out of seven in a combined analysis of 13 CHARGE cohorts imputed with 1000 Genomes Phase 1. All effect estimates and allele frequencies are with respect to the coded allele

**Figure 1 (Box 1):**
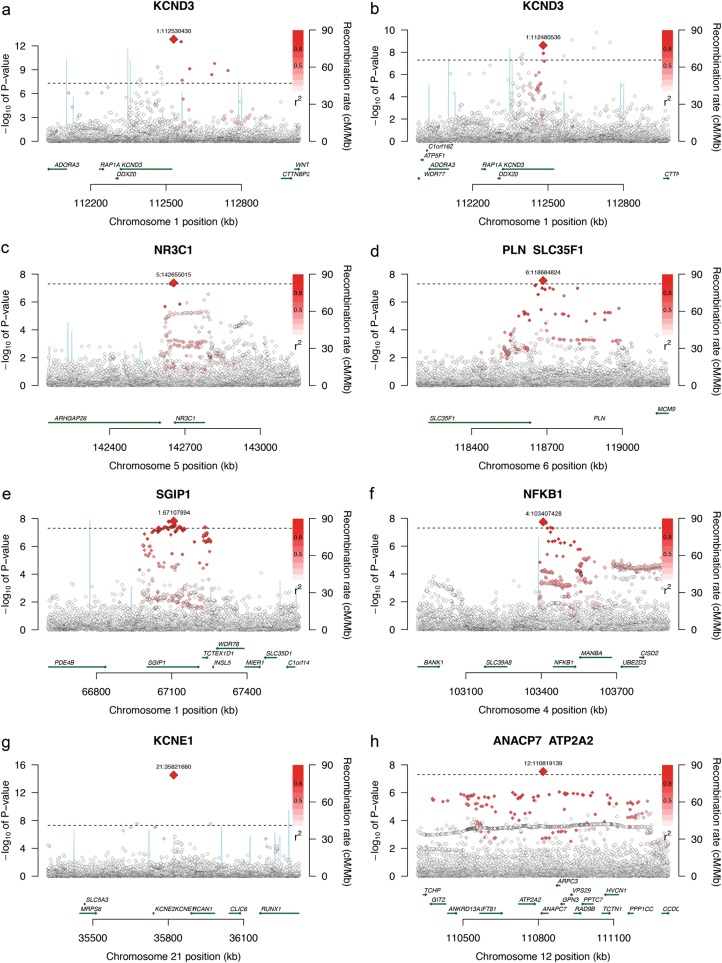
Novel loci associated with PR, QRS, and QT. *KCND3*, associated with PR interval (**a**, **b**). *ARHGAP26* and *NR3C1*, associated with PR interval (**c**). *SLC35F1* and *PLN*, associated with PR interval (**d**). *ATP2A2*, associated with QRS duration (**e**). *SGIP1* and *TCTEX1D1*, associated with QT interval (**f**). *NFKB1*, associated with QT interval (**g**). *KCNE1*, associated with QT interval (**h**)

Box 1 Seven novel loci were identified; three for PR, three for QT, and one for QRS. Information and regional association plots are shown for every locus. Each SNP is plotted with respect to its chromosomal location (hg19, *x*-axis) and its *P*-value (*y*-axis on the left). The tall blue spikes indicate the recombination rate (*y*-axis on the right) at that region of the chromosome.

We observed two independent signals at the *KCND3* gene. The first signal consists of low-frequency SNPs (MAF < 3.8%, index SNP MAF = 2.4%) upstream of *KCND3* (top), while the second signal contains intronic SNPs with much higher allele frequencies (index SNP MAF = 19.6%, bottom). *KCND3* encodes voltage-gated potassium channel subunit K_v_4.3. SNPs near *KCND3* have been associated with P-wave duration and ST-T wave amplitude [29], and with Atrial Fibrillation in the Japanese population [30]. It is thought that *KCND3* overexpression may be involved in Brugada syndrome because of its direct interaction with *KCNE3*. This gene inhibits *KCND3*, and specific mutations in the latter gene lead to Brugada syndrome [31, 32]. Moreover, it has been shown that mutations in *KCND3* cause spinocerebellar ataxia [33] (Fig. 1a, b).

The association signal in this locus spans the *NR3C1* gene, with the two genome-wide significant SNPs located between *NR3C1* and *ARHGAP26*. Both SNPs are common, with MAFs of approximately 45%. *NR3C1* encodes the glucocorticoid receptor, which interacts with a wide variety of proteins, transcription factors, and other cellular compounds [34]. In mice, this gene is involved in cardiac development [35], and overexpression causes ECG abnormalities [36], which makes it likely that this is the gene underlying the association signal. *ARHGAP26* encodes GRAF protein (GTPase Regulator Associated with Focal Adhesion Kinase), which is required in specific exo- and endocytosis pathways [37], but also for muscle development [38]. Mutations in this gene have been implicated in leukemia [39] (Fig. 1c).

Fig 1d: This locus has been associated previously with RR interval [4], QT interval [8, 9], and QRS duration [7]. The index SNP has a MAF of 5.4% and the association signals spans *SLC35F1* and *PLN*. The latter gene encodes phospholamban, which is an important regulator of cardiac contractility [40]. *SLC35F1* encodes a transporter protein that is highly expressed in the human brain [41] (Fig. 1d).

Although only one (common, MAF = 32.2%) SNP reached genome-wide significance, SNPs in strong LD with the index SNP span an area of almost 500 kb, covering many genes. This locus has been associated with QT interval previously [10]. Our most significant SNP is located just downstream of *ATP2A2*, a strong candidate gene in this region that encodes a SERCA Ca^2+^ ATPase, which is involved in calcium transport in the human heart and under regulation of phospholamban [42] (Fig. 1e).

This locus spans ~300 kb in between two recombination hotspots. Significant SNPs are in almost complete LD with each other, with minor allele frequencies of approximately 15%. The locus spans two genes, *SGIP1* and *TCTEX1D1*. *SGIP1* encodes a proline-rich endocytic protein that interacts with endophilin and is involved in energy homeostasis [43, 44]. This gene is mainly expressed in the human brain [43] and has been associated with fat mass [45]. The *TCTEX1D1* gene belongs to the dynein light chain Tctex-type family and has an unknown function (Fig. 1f).

The most significant SNPs in this locus are located upstream of the *NFKB1* gene, encoding the NF-kappa-B p105 subunit. SNPs in this locus are common (MAF = 43.5%). An indel in the promotor of this gene has been associated with coronary heart disease [46] and dilated cardiomyopathy [47]. This particular indel is in moderate LD with the index SNP in this locus (*r*^2^ in GoNL = 0.4). *NFKB1* is a transcription factor is involved in many immune- and tumor-related processes, and has been associated with ulcerative colitis [48] and bladder cancer [49] (Fig. 1g).

This locus contains a low frequency SNP (MAF = 1.7%) with a large effect on QT interval. This SNP has been observed in GWAS before, but could not be replicated (in this [8] and later studies [10]) because it was poorly imputed so only cohorts that genotyped the SNP directly could be included [8]. *KCNE1* encodes a voltage-gated potassium channel, and the index SNP encodes a pathogenic Asp to Asn amino acid substitution at position 85 of *KCNE1*, causing long QT syndrome 5 [50] (Fig. 1h).

### Fine-mapping of known loci

For each locus, we tested if more than one independent signal was present (Supplementary Table 4). Thirteen loci had suggestive evidence of having more than one independent signal; four locus-phenotype associations had five or more independent signals. The *SCN5A*/*SCN10A* locus was the most outstanding locus with eleven independent signals for PR, and six for QRS. *NOS1AP* for QT contained seven independent signals.

### Replication in CHARGE

For six out of seven novel loci, we were able to conduct look-ups of the index SNP or a proxy SNP in strong LD (*r*^2^ ≥ 0.89) in previous large-scale HapMap-based GWAS. These GWAS contained over 70,000 samples each, and included many of the Dutch cohorts from our current study. All six loci were associated with their respective traits (*P* ≤ 0.004). Next, we tested the seven novel loci for replication in 13 studies from the CHARGE consortium. In contrast to the HapMap look-ups, this replication was independent from the Dutch discovery sample. Results are shown in Table 1. Allele frequencies were very similar to the discovery dataset, except for JHS, which consists of individuals of African-American descent. Effect directions for all seven SNPs were concordant between our primary findings and replication, with effect sizes between 0.2 and 1.5 times those of the betas in the discovery study. Six of seven loci were replicated with *P* < 0.05, three of which pass Bonferroni correction, accounting for seven tests.

### Functional SNPs in genes and regulatory regions

All genome-wide significant SNPs were tested in silico for their potential effect on gene expression and protein structure. Ten loci contained, in total, 15 non-synonymous SNPs, which were tested using the prediction programs PolyPhen-2 and SIFT. According to PolyPhen-2, three SNPs were possibly damaging (rs1805128 in *KCNE1* for QT, rs12666989 in *UFSP1* for RR, and rs2070492 in *SLC22A14* for PR). SIFT predicted only one SNP to be damaging to a protein (rs3746471 in *KIAA1755* for RR).

We used GREAT to test all 100 index SNPs from the four ECG traits combined for their biological function in *cis*-regulatory regions. Significant GO-terms (molecular function, biological process, and cellular component), human phenotypes, and disease ontologies are shown in Supplementary Table 5a–d. In total, these index SNPs mapped to 103 genes.

Of 52 locus-phenotype associations, 34 contained significant SNPs that have a RegulomeDB score of 3 or better, meaning that they may affect protein binding (Supplementary Table 6). We observed 15 loci containing SNPs with scores of 1 (likely to affect binding and linked to the expression of a gene target), 15 loci containing SNPs with a maximum score of 2 (likely to affect binding), and four loci that have SNPs with a maximum score of 3 (less likely to affect binding). Eighteen loci contained only SNPs with scores from 4 to 6 (minimal binding evidence) and 7 (no data available).

## Discussion

We imputed over 19 million SNPs using GoNL as the reference panel, and tested these SNPs for association with four traits in eight predominantly Dutch cohorts comprising roughly 30,000 samples. We observed 52 locus-phenotype associations, seven of which were novel (Table 1, Box 1, Supplementary Table 4).

### Discovery of loci associated with quantitative ECG traits

We detected seven novel loci, three for PR interval, three for QT interval, and one for QRS duration (Box 1). No novel loci were found for RR interval, accounting for loci previously associated with either RR interval [4] or heart rate [5]. We replicated six out of seven novel loci utilizing 13 independent studies from the CHARGE consortium. Interestingly, the only variant that does not replicate is rs74640693 for PR interval, located close to *PLN* (phospholamban). Variants in this gene have been consistently associated with various QRS measures [6] but not with PR interval. The gene transcribes the phospholamban protein, which is important in calcium signaling in cardiac muscle cells [24]. Although a Dutch-specific pathogenic mutation, p.Arg14del, in the *PLN* gene has been described [25], it is unlikely that this mutation drives the association signal in our study because the allele frequency of SNP rs74640693 is similar in our samples (4.9%) compared to other samples of European ancestry (4.6% in the 12 European CHARGE replication cohorts). Furthermore, the allele frequency of this SNP is ~5 times higher than that of the mutation and the SNP is located ~200 kb upstream of the PLN gene, so, therefore, not in LD with these mutations. In addition, a recent large study of PR interval used the Illumina exome chip to identify a common variant (rs74640693, allele frequency 47%) in this region [26], however, this variant is not in LD with the variant that we identified (*r*^2^ = 0.04). To confirm that the lack of association was not caused by strand issues (because rs74640693 is an A/T variant), we tested the nearby proxy SNP rs12210733 (which is an A/G variant, *r*^2^ = 0.89) in the CHARGE replication cohorts, and found it was also non-significant.

We looked up our top SNPs in previous, much larger, HapMap-based GWAS meta-analyses to determine why our SNPs were not identified in those studies (Table 1). Two loci contained rare SNPs with MAF < 5%. Low-frequency SNPs at *KCND3* were not present in HapMap and could therefore not be tested. The functional SNP at *KCNE1* was observed in a single cohort in a meta-analysis in 2009, but this result could neither be replicated in other cohorts [9], nor in later studies, because the imputation quality was too low.

For common SNPs (MAF > 5%), it is much more difficult to define why they were not previously observed at genome-wide significance. For many loci we may have better tags of the causal variants because our coverage is almost sevenfold greater. Indeed, the index SNPs at *PLN* (PR), *NFKB1* (QT), and *ATP2A2* (QRS) were not tested in previous studies. Nevertheless, for all SNPs, proxies with *r*^2^ > 0.9 were available in the respective studies (Table 1). Common SNPs at *KCND3* (PR), *NR3C1* (PR), and *SGIP1* (QT) were present in HapMap. Both proxies and directly imputed SNPs were at least nominally significant in previous studies (*P*-values ranging from 10^–3^ to 10^–6^) with typically high imputation quality.

In addition to the “winner’s curse” effect, we expect that higher quality imputation due to the considerably larger haplotype panel (compared to HapMap) and the ancestry matching between GoNL and our Dutch cohorts will improve the power to detect a true association signal, if present. Although combining multiple reference panels for imputation is becoming the new standard [27], limitations to our study remain: (1) the GoNL reference panel may not contain sufficient information on rare SNPs; (2) the small sample size of individual cohorts may cause abnormal behavior of rare SNPs as a group, requiring us to remove that subset of SNPs; or (3) the sample size or power of the overall study is still limited to detect rare variant associations.

### Fine-mapping of known loci

Although we did not sequence the loci containing the known and novel signals, we have a much denser interrogation of these regions compared to previous (HapMap-based) studies. In an attempt to fine map the significant loci, we annotated all significant SNPs with their predicted functional consequences.

First, we used SIFT and PolyPhen-2 to predict the effect of 15 non-synonymous SNPs that were associated with one of the ECG traits at genome-wide significance. PolyPhen-2 classified three SNPs as possibly damaging and SIFT predicted only one SNP to be damaging. These were non-overlapping, raising questions as to the actual effect of these SNPs on their respective genes. Functional studies should be pursued to test the direct effect of these SNPs on protein structure.

Combining all index SNPs, we tested the function of those SNPs located in *cis*-regulatory regions using GREAT [22]. We identified 100 independent SNP-trait associations, which mapped to 103 genes. Interestingly, we find hundreds of significant nodes, of which the vast majority is related to cardiac functioning and heart disease (Supplementary Table 5a–e). This shows that, indeed, many SNPs are located in *cis*-regulatory regions of genes that are critical in the functioning of the human heart, which implicates a regulatory function of these loci rather than a structural function changing the protein directly. One example is shown in Supplementary Figure 3; this figure contains all significant GO molecular function nodes. Most of these nodes are in the group of transporter activity, which includes all transmembrane channels that are known to be important for cardiac function.

Because the GREAT pathways show that many SNPs probably have their effect on the trait due to gene regulation, we extracted all significant SNPs from RegulomeDB to check which variants would likely affect binding in regulatory regions. A majority of loci contained at least one SNP that was expected to affect transcription factor binding (Supplementary Table 6). The score that is provided by RegulomeDB indicates that a SNP is likely (or less likely) located in a binding site. Interestingly, there are strong differences between loci in terms of the number of SNPs that may have a regulatory effect, and percentage of loci per trait that have a high score. For instance, seven out of 15 QT interval loci contains SNPs with a score of 1, while only a single PR interval locus contains a SNP with this score. The *SCN5A*/*SCN10A* locus is strongly associated with PR interval (best SNP *P* = 4.9 × 10^–107^) and contains over 450 significant SNPs. Nevertheless, only six SNPs have a score of 2 or 3, and none of the significant SNPs have a score of 1. However, many binding sites are tissue specific, and, therefore, testing SNPs with high scores systematically for their role in cardiac tissue could lead to more knowledge about their biological function.

## Conclusions

Using the Genome of the Netherlands as imputation reference panel, we identified seven novel loci for quantitative ECG traits. Higher SNP density and higher imputation quality enabled us to annotate known loci, facilitating future studies to understand the biological effects of causal variants at many loci. Ultimately, combining imputation reference panels and increasing sample size for GWAS meta-analyses will continue to increase power for genetic discovery and novel target identification. With many sequencing efforts ongoing and large population-based cohorts being genotyped (such as UK Biobank, of which the first release data showed 46 novel loci for RR interval [28]), we can expect hundreds of novel loci for ECG phenotypes in the near future.

## Electronic supplementary material

Supplementary material
