# Supplementary material for: Genome-wide association meta-analysis of 30,000 samples identifies seven novel loci for quantitative ECG traits
Source: Eur J Hum Genet. 2019 Jan 24;27(6):952–62. doi: 10.1038/s41431-018-0295-z (PMC6777533; doi:10.1038/s41431-018-0295-z)
Supplement: Supplementary file 1 — Supplementary material [file 41431_2018_295_MOESM1_ESM.docx]

**Supplementary material**

**GWAS meta-analysis of 30,000 samples identifies seven novel loci for quantitative ECG traits.**

Jessica van Setten, Niek Verweij, Hamdi Mbarek, Maartje N. Niemeijer, Stella Trompet, Dan E. Arking, Jennifer A. Brody, Ilaria Gandin, Niels Grarup, Leanne M. Hall, Daiane Hemerich, Leo-Pekka Lyytikäinen, Hao Mei, Martina Müller-Nurasyid, Bram P. Prins, Antonietta Robino, Albert V. Smith, Helen R. Warren, Folkert W. Asselbergs, Dorret I. Boomsma, Mark J. Caulfield, Mark Eijgelsheim, Ian Ford, Torben Hansen, Tamara B. Harris, Susan R. Heckbert, Jouke-Jan Hottenga, Anita Iorio, Jan A. Kors, Allan Linneberg, Peter MacFarlane, Thomas Meitinger, Christopher P. Nelson, Olli T. Raitakari, Claudia T. Silva Aldana, Gianfranco Sinagra, Moritz Sinner, Elsayed Z. Soliman, Monika Stoll, Andre Uitterlinden, Cornelia M. van Duijn, Melanie Waldenberger, Alvaro Alonso, Paolo Gasparini, Vilmundur Gudnason, Yalda Jamshidi, Stefan Kääb, Jørgen K Kanters, Terho Lehtimäki, Patricia B. Munroe, Annette Peters, Nilesh J. Samani, Nona Sotoodehnia, Sheila Ulivi, James G. Wilson, Eco J.C. de Geus, J. Wouter Jukema, Bruno Stricker, Pim van der Harst, Paul I.W. de Bakker, Aaron Isaacs

**Contents:**

Cohort information 3

Supplementary Figure 1 10

Supplementary Figure 2 11

Supplementary Figure 3 13

Supplementary Table 1 15

Supplementary Table 2 16

Supplementary Table 3 20

Supplementary Table 4 21

Supplementary Table 5 25

Supplementary Table 6 38

References 42

Funding 44

Acknowledgments 48

**Cohort information**

**Discovery cohorts**

**ERF (Erasmus Rucphen Family Study)**

The Erasmus Rucphen Family study^1^ is comprised of a family-based cohort embedded in the Genetic Research in Isolated Populations (GRIP) program in the southwest of the Netherlands. The aim of this program is to identify genetic risk factors for the development of complex disorders. In ERF, twenty-two families that had a large number of children baptized in the community church between 1850 and 1900 were identified with the help of detailed genealogical records. All living descendants of these couples, and their spouses, were invited to take part in the study. Comprehensive interviews, questionnaires, and examinations were completed at a research center in the area; approximately 3,200 individuals participated. Examinations included 12 lead ECG measurements. Electrocardiograms were recorded on ACTA electrocardiographs (ESAOTE, Florence, Italy) and digital measurements of the PR, QRS, QT and RR intervals were made using the Modular ECG Analysis System (MEANS). Data collection started in June 2002 and was completed in February 2005. In the current analyses, 2442 participants for whom complete phenotypic, genotypic and genealogical information was available were studied.

Study design: family-based cohort

ECG recording information: [A 10-second 12-lead ECG was recorded with an ACTA-ECG electrocardiograph (Esaote, Florence, Italy) with a sampling frequency of 500 Hz. Digital measurements of the ECG parameters were made using the Modular ECG Analysis System (MEANS).](#RANGE!_ENREF_56)

**Lifelines (LifeLines Cohort Study & Biobank)**

LifeLines^2^ is a multi-disciplinary prospective population-based cohort study examining in a unique three-generation design the health and health-related behaviours of 165,000 persons living in the North East region of The Netherlands. It employs a broad range of investigative procedures in assessing the biomedical, socio-demographic, behavioural, physical and psychological factors which contribute to the health and disease of the general population, with a special focus on multimorbidity and complex genetics. Details of the protocol have been described elsewhere (https://www.lifelines.nl/lifelines-research/news). Standard 12-lead electrocardiograms were recorded with CardioPerfect equipment (Cardio Control; currently Welch Allyn, Delft, The Netherlands) and digital measurements of the QT intervals were extracted.

Study design: Population based

ECG recoding information: Standard 12 lead, Cardio Perfect equipment (Welch Allyn Cardio Control, Delft, The Netherlands)

**NTR (The Netherlands Twin Register)**

NTR^3, 4^ participants are ascertained because of the presence of twins or triplets in the family and consist of multiples, their parents, siblings and spouses. Twins are born in all strata of society and NTR represents a general sample from the Dutch population.

Study design: Twin registry

ECG recording information: Resting ECG in sitting position for 4-8 minutes; 3-lead ECG – ECG100C module, Biopac Systems or 3-lead ECG (same type II configuration) VU-AMS5fs, Vrije Universiteit Amsterdam

**Prevend (Prevention of Renal and Vascular End-Stage Disease)**

Prevend^5^ is a prospective study investigating the natural course of increased levels of urinary albumin excretion and its relation to renal and cardiovascular disease. Details of the protocol have been described elsewhere (www.prevend.org)

Study design: Population based

ECG recoding information: Standard 12 lead, Cardio Perfect equipment (Welch Allyn Cardio Control, Delft, The Netherlands)

**PROSPER (PROspective Study of Pravastatin in the Elderly at Risk)**

PROSPER^6-8^ includes individuals >70 year, with an increased risk of cvd.

Study design: RCT/population based

ECG recording information: 12 Lead ECG

**RS I, RS II, RS III (Rotterdam Study Cohort I, Rotterdam Study Cohort II, Rotterdam Study Cohort III)**

The Rotterdam Elderly Study^9^ is a prospective cohort study in the Ommoord district in the city of Rotterdam, the Netherlands [Hofman et al., 1991]. Following the pilot in 1989, recruitment started in January 1990. The main objectives of the Rotterdam Study were to investigate the risk factors of cardiovascular, neurological, ophthalmological and endocrine diseases in the elderly. Up to 2008, approximately 15,000 subjects aged 45 years or over have been recruited. Participants were interviewed at home and went through an extensive set of examinations, bone mineral densiometry, including sample collections for in-depth molecular and genetic analyses. Examinations were repeated every 3-4 years in potentially changing characteristics. Participants were followed for the most common diseases in the elderly, including coronary heart disease, heart failure and stroke, Parkinson's disease, Alzheimer's disease and other dementias, depression and anxiety disorders, macular degeneration and glaucoma, diabetes mellitus and osteoporosis.

Study design: Population based

ECG recoding information: ECG device: EACTA, ASOTE, florence, italy; software: MEANS

**Replication cohorts (CHARGE consortium)**

**AGES (Age, Gene/Environment Susceptibility Reykjavik Study)**

In anticipation of the sequencing of the human genome and description of the human proteome, the Age, Gene/Environment Susceptibility-Reykjavik Study (AGES-Reykjavik)^10^ was initiated in 2002. AGES-Reykjavik was designed to examine risk factors, including genetic susceptibility and gene/environment interaction, in relation to disease and disability in old age. The study is multidisciplinary, providing detailed phenotypes related to the cardiovascular, neurocognitive (including sensory), and musculoskeletal systems, and to body composition and metabolic regulation. Relevant quantitative traits, subclinical indicators of disease, and medical diagnoses are identified by using biomarkers, imaging, and other physiologic indicators. The AGES-Reykjavik sample is drawn from an established population-based cohort, the Reykjavik Study. This cohort of men and women born between 1907 and 1935 has been followed in Iceland since 1967 by the Icelandic Heart Association. The AGES-Reykjavik cohort, with cardiovascular risk factor assessments earlier in life and detailed late-life phenotypes of quantitative traits, will create a comprehensive study of aging nested in a relatively genetically homogeneous older population. This approach should facilitate identification of genetic factors that contribute to healthy aging as well as the chronic conditions common in old age.

Study design: Population based

ECG recording information: Marquette 12SL

**ARIC (Atherosclerosis Risk in Communities)**

ARIC^11^ is a prospective epidemiologic study conducted in four U.S. communities. ARIC is designed to investigate the etiology and natural history of atherosclerosis, the etiology of clinical atherosclerotic diseases, and variation in cardiovascular risk factors, medical care and disease by race, gender, location, and date.

Study design: Population-based

ECG recording information: The study ECGs were recorded with MAC PC ECG machines (Marquette Electronics, Milwaukee, WI) in all clinical centers. ECGs were initially processed in a central laboratory at the EPICORE Center (University of Alberta, Edmonton, Alberta, Canada) and during later phases of the study at the EPICARE Center (Wake Forest University, Winston-Salem, NC). All ECGs were visually inspected for technical errors and inadequate quality. Initial ECG processing was done by the Dalhousie ECG program, and processing was later repeated with the 2001 version of the GE Marquette 12-SL program (GE Marquette, Milwaukee, WI).

**BRIGHT (British Genetics of Hypertension)**

Hypertensive Cases: White Europeans from UK^12^

Study design: Hypertensive cases

ECG recording information: Twelve-lead ECG recordings (Siemens-Sicard 440)

**CHS (Cardiovascular Health Study)**

The CHS^13^ is a population-based cohort study of risk factors for coronary heart disease and stroke in adults ≥65 years conducted across four field centers. The original predominantly European ancestry cohort of 5,201 persons was recruited in 1989-1990 from random samples of the Medicare eligibility lists; subsequently, an additional predominantly African-American cohort of 687 persons was enrolled for a total sample of 5,888.

Study-design: Cohort study

ECG recording information: Twelve-lead resting electrocardiographs with standard lead placements were recorded during ten seconds at baseline using Marquette MAC PC-DT electrocardiographic recorder (Marquette Electronics Inc, Milwaulkee, Wisconsin).

**GRAPHIC**

The GRAPHIC^14^ Study comprises 2024 individuals from 520 nuclear families recruited from the general population in Leicestershire, UK between 2003-2005 for the purpose of investigating the genetic determinants of blood pressure and related cardiovascular traits. Families were included if both parents aged 40-60 years and two offspring ≥18 years wished to participate. A detailed medical history was obtained from study subjects by standardized questionnaires and clinical examination was performed by research nurses following standard procedures. Measurements obtained included height, weight, waist-hip ratio, clinic and ambulatory blood pressure and a 12-lead ECG.

Study design: Population based

ECG recording information: Standard 12-lead ECGs were recorded on either Siemens 460 electrocardiographs or Burdick Eclipse or Atria models. Digital ECG data were transferred to the University of Glasgow ECG Core Lab based in Glasgow Royal Infirmary and ECGs were analysed by the University of Glasgow ECG analysis program.

**INGI-Carlantino**

The Carlantino cohort (INGI-CARL)^15^ includes approximately 1000 samples from an isolated village of Southern Italy

Study design: Isolated population

ECG recording information: Digital caliper measurements were made on scanned paper ECGs recorded at 25 mm/sec. Mortara instrument ELI 250 was used to obtain ECG measurements.

**INGI-FVG (INGI-FRIULI VENEZIA GIULIA)**

INGI-FVG^15^ is characterized by approximately 1700 samples from six isolated villages of Northern Italy

Study design: Isolated population

ECG recording information: Digital caliper measurements were made on scanned paper ECGs recorded at 25 mm/sec. Mortara instrument ELI 250 was used to obtain ECG measurements.

**Inter99**

The Inter99^16^ was study carried out in 1999-2001 included invitation of 12934 persons aged 30-60 years drawn from an age- and sex-stratified random sample of the population. The baseline participation rate was 52.5%, and the study included 6784 persons. The Inter99 study was a population-based randomized controlled trial (CT00289237, ClinicalTrials.gov) and investigated the effects of lifestyle intervention on CVD. Here 5951 participants with information on ECG and exome chip were analysed.

Study design: Population-based

ECG recording information: MUSE Cardiology Information System (GE Healthcare, Wauwatosa, Wisconsin) analysed by Marquette 12SL algorithm version 21.

**JHS (Jackson Heart Study)**

The JHS^17^ is a single-site cohort study of 5,301 extensively phenotyped African American women and men. Three clinical examinations have been completed, including the baseline examination, Examination 1 (2000–2004), Examination 2 (2005–2008), and Examination 3 (2009–2013), allowing comprehensive assessment of cardiovascular health and disease of the cohort at approximately four-year intervals. Ongoing monitoring of hospitalizations for cardiovascular events (coronary heart disease, heart failure and stroke) and deaths among cohort participants are accomplished by annual telephone follow-up interviews, surveillance of hospital discharge records (since 2000 for coronary heart disease and stroke, and since 2005 for heart failure), and vital records.

Study design: Single-site observational cohort study with a nested family cohort.

ECG recording information: A supine 12-lead digital electrocardiogram (ECG) was recorded with the Marquette MAC/PC digital ECG recorder (Marquette Electronics, Milwaukee, WI), and with electrode placement that duplicates that of the ARIC study. The ECGs are analyzed in accordance with the Minnesota Code Classification system. In-hospital surveillance ECGs are read visually according to the Minnesota Code Classification system.

**KORA F3** and **KORA S4 (Kooperative Gesundheitsforschung in der Region Augsburg)**

The KORA Study^18, 19^ is a series of population-based epidemiological surveys of persons living in Augsburg, Southern Germany, or its two adjacent counties. All survey participants are residents of German nationality identified through the registration office and between 25 and 75 years old at the time of enrollment. The KORA S4 study is a baseline survey that was conducted in the years 1999-2001.

Study design: Population based

ECG recording information: 12-lead resting electrocardiograms were recorded with digital recording systems (F3: Mortara Portrait, Mortara Inc., Milwaukee, USA, S4: Hörmann Bioset 9000, Hörmann Medizinelektronik,Germany).

**TwinsUK**

TwinsUK^20^ is a nation-wide registry of volunteer twins in the United Kingdom, with about 12,000 registered twins (83% female, equal number of monozygotic and dizygotic twins, predominantly middle-aged and older). Over the last 20 years, questionnaire and blood/urine/tissue samples have been collected on over 7,000 subjects, as well as three comprehensive phenotyping assessments in the clinical facilities of the Department of Twin Research and Genetic Epidemiology, King's College London. The primary focus of study has been the genetic basis of healthy aging process and complex diseases, including cardiovascular, metabolic, musculoskeletal, and ophthalmologic disorders. Alongside the detailed clinical, biochemical, behavioral, and socio-economic characterization of the study population, the major strength of TwinsUK is availability of several 'omics' technologies for the participants. These include genome-wide scans of single nucleotide variants, next-generation sequencing, exome sequencing, epigenetic markers (MeDIP sequencing), gene expression arrays and RNA sequencing, telomere length measures, metabolomic profiles, and gut flora microbiomics.

Study design: Twin registry

ECG recording information: 12-lead ECG- Cardiofax GEM machine 9020K (1997 onward), Phillips Page Writer Trim l Cardiograph ECG machine (2009 onward)

**YFS (The Cardiovascular Risk in Young Finns Study)**

The YFS^21^ is a population-based follow up-study started in 1980. The main aim of the YFS is to determine the contribution made by childhood lifestyle, biological and psychological measures to the risk of cardiovascular diseases in adulthood. In 1980, over 3,500 children and adolescents all around Finland participated in the baseline study. The follow-up studies have been conducted mainly with 3-year intervals. The latest 30-year follow-up study was conducted in 2010-11 (ages 33-49 years) with 2,063 participants. The study was approved by the local ethics committees (University Hospitals of Helsinki, Turku, Tampere, Kuopio and Oulu) and was conducted following the guidelines of the Declaration of Helsinki. All participants gave their written informed consent.

Study design: Population based

ECG recording information: In 2001, a single channel ECG was recorded during a 3-minute period. The ECG signal was collected after the participants had remained comfortably in a supine position for at least 15 minutes. The three ECG leads were positioned diagonally as follows: 1) above sternum, 2) below sternum 3) above umbilicus. The resulting QRS complex corresponds to leads V1-V2. All ECG signals were examined visually by one operator.


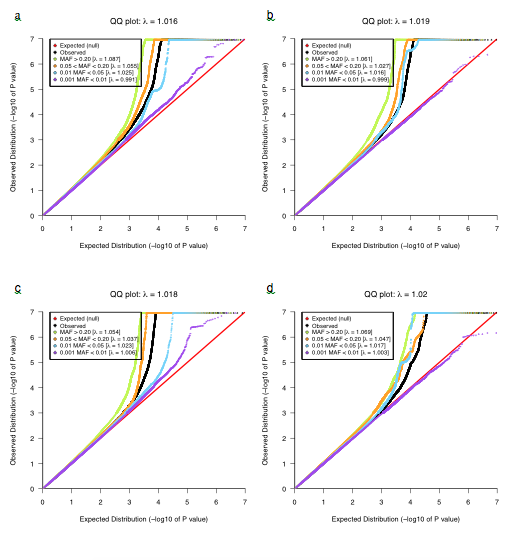


**Supplementary Figure 1: Quantile-quantile plots of four GWAS meta-analyses.** These plots show the observed versus expected distribution of *P*-values of meta-analysis results of PR interval (a), QRS duration (b), QT interval (c), and RR interval (d). The red line represents the null (expected) distribution, while the black dots show the observed distribution of all P-values. Green, orange, blue, and purple dots depict SNPs with minor allele frequency above 20%, between 5% and 20%, between 1% and 5%, and between 1% and 0.1%, respectively. SNPs with allele frequencies above 1% show clear deviation from the null for all four traits. All traits are predicted to be highly polygenic and this deviation indicates many additional loci that may be involved. The allele frequency bins below 1% have less power to detect associations and follow the null distribution, and lambda (λ) < 1.02 for all traits, suggesting that population stratification is minimal.


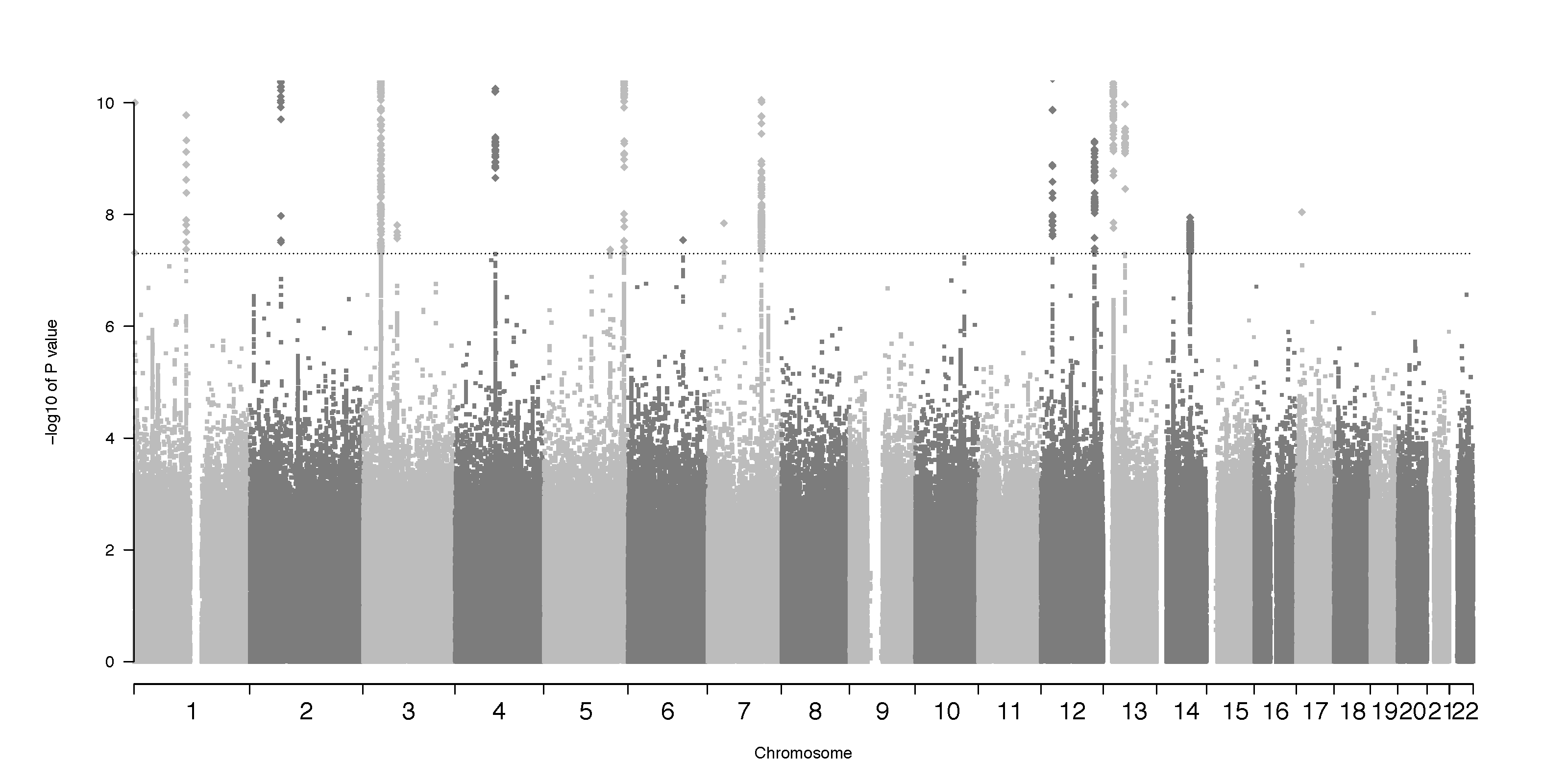


a

**Supplementary Figure 2: Genome-wide results of four quantitative ECG traits in 30,000 individuals of European descent.** Nineteen million SNPs were tested for association. The Manhattan plots show the meta-analysis association results for PR interval (a), QRS duration (b), QT interval (c), and RR interval (d). The dashed line marks the genome-wide significant level of 5 x 10^-8^. The Y-axis is truncated at *P* = 1 x 10^-10^.


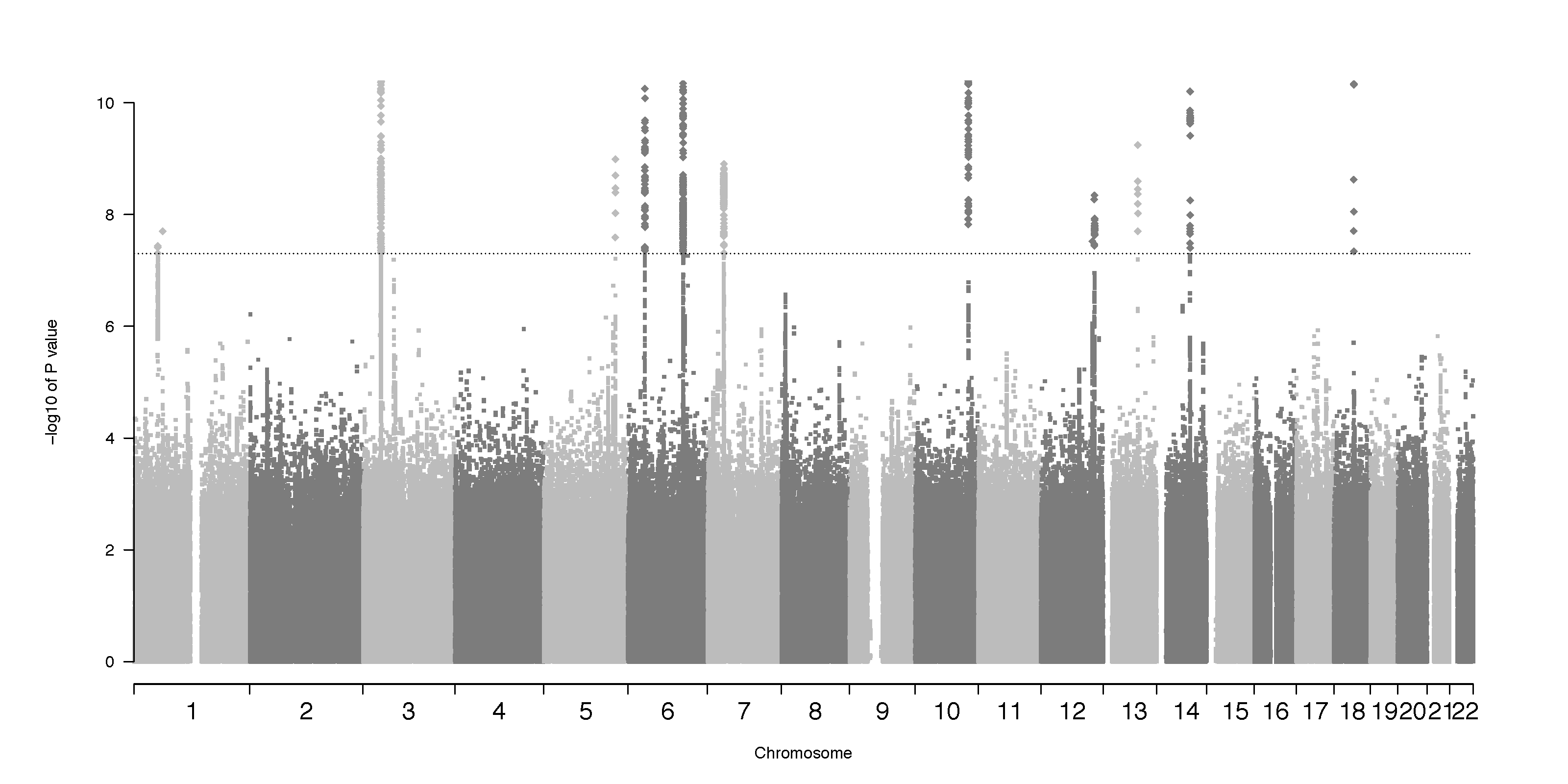


b


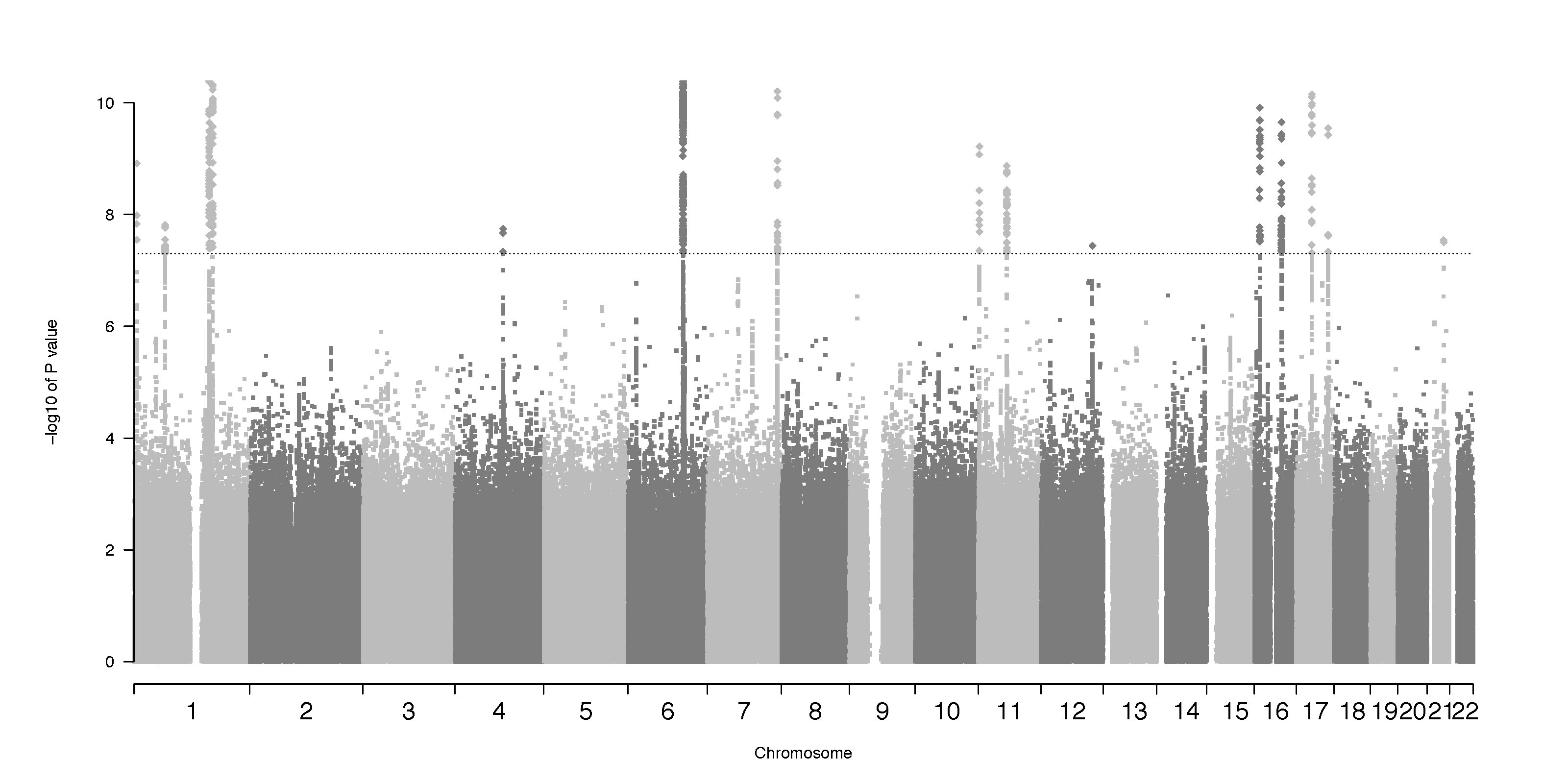


c


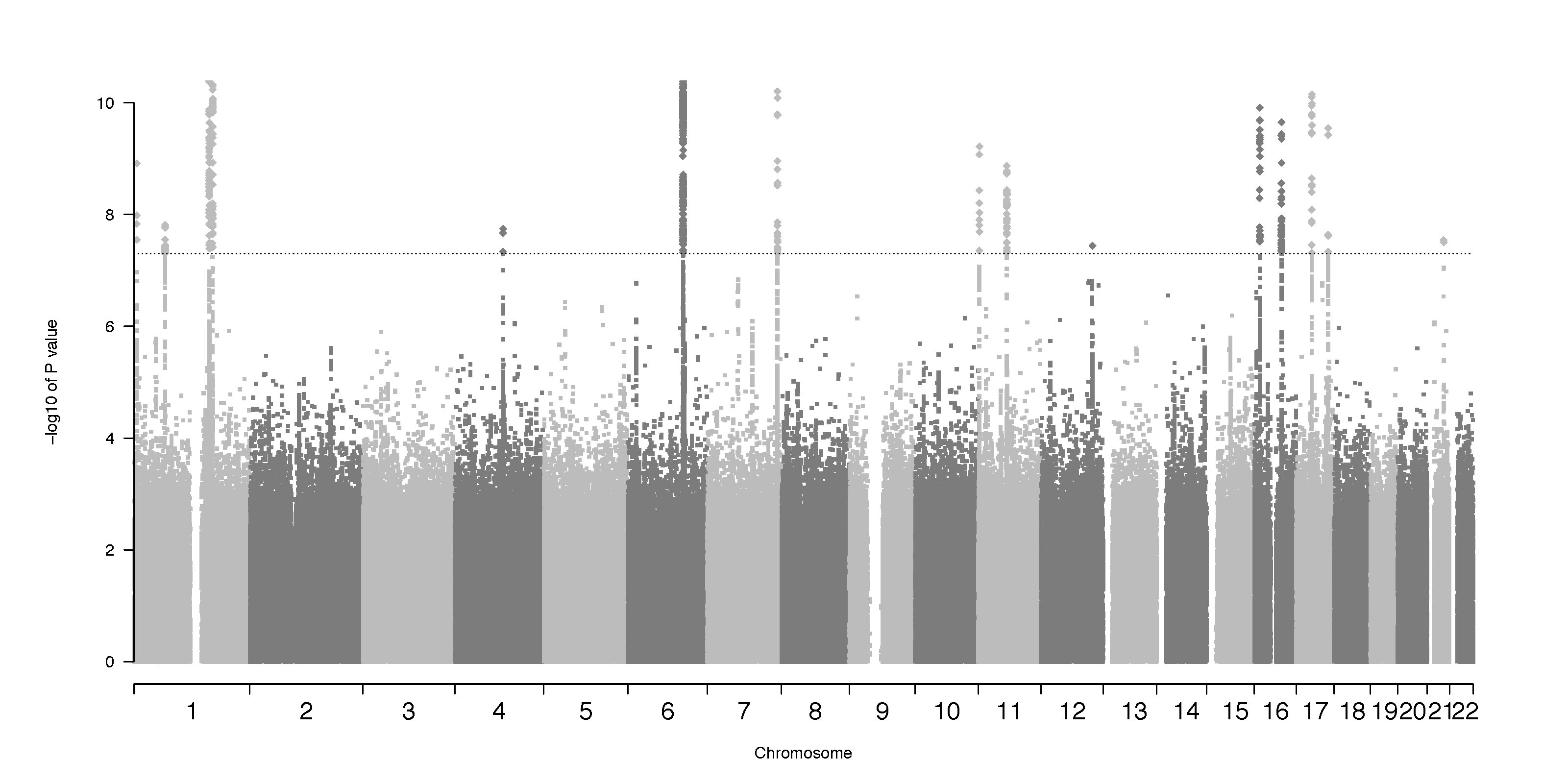


d

**Next page: Supplementary Figure 3: Pathways involved in PR interval. QRS duration, QT interval, and RR interval.** All 100 independent locus-trait associated SNPs were combined and mapped to genes based on *cis*-regulatory regions. Using GREAT,^22^ enriched nodes were identified and combined into pathways. This figure shows all significant GO Molecular function nodes, most of which are in the same pathway. Significant nodes (FDR correction) are shown in blue, the numbers indicate fold-enrichment.


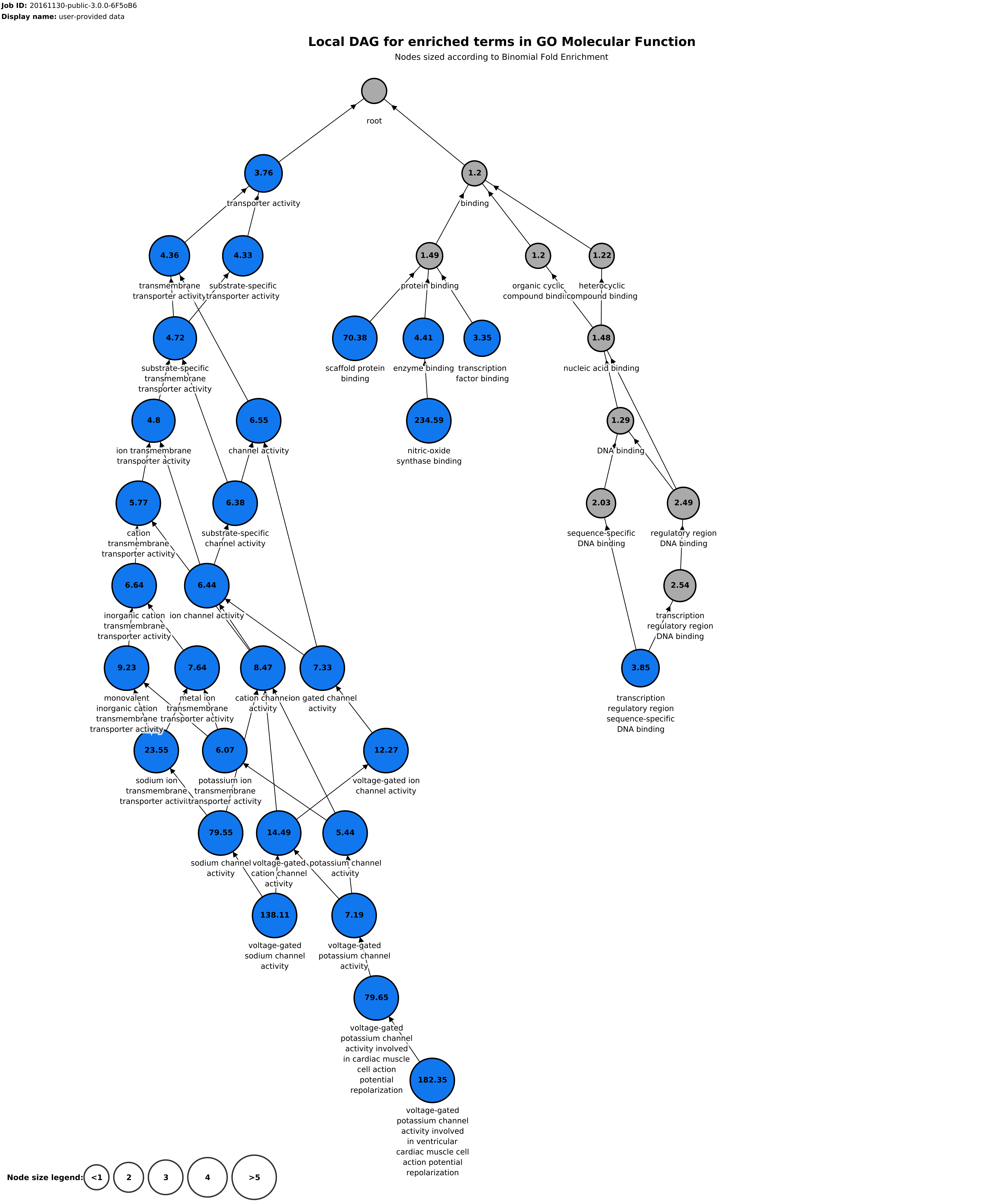


**Supplementary Table 1: Phenotype characteristics**

| COHORT | PR interval | | QRS duration | | QT interval | | RR interval | | Other | | | | |
| --- | --- | --- | --- | --- | --- | --- | --- | --- | --- | --- | --- | --- | --- |
|  | Sample size (n) | Mean PR interval (sd) | Sample size (n) | Mean QRS duration (sd) | Sample size (n) | Mean QT interval (sd) | Sample size (n) | Mean RR interval (sd) | Mean age (sd) | Mean height (sd) | Mean BMI (sd) | n Females (%) | Ancestry |
| AGES | 2378 | 170.9 (26.8) | 2280 | 90.3 (10.6) | 2540 | 405.9 (34.8) | N/A | N/A | 76.2 (5.4) | 166.7 (9.2) | 27.1 (4.5) | 60.30% | European |
| ARIC | 8038 | 160.2 (23.1) | 8040 | 91.1 (9.5) | 8040 | 398.7 (28.5) | N/A | N/A | 54 (5.7) | 168.6 (9.4) | 26.8 (4.7) | 4360 (54.2) | European |
| BRIGHT | 1186 | 162.4 (19.5) | 1229 | 93.0 (9.4) | 1326 | 422.1 (26.5) | N/A | N/A | 39.9 (11.0) | 166 (9) | 27.5 (3.8) | 819 (61.8) | European |
| Carlantino | 391 | 151.4 (21.8) | 391 | 91.3 (13.5) | 391 | 390.5 (28.5) | N/A | N/A | 44.6 (20.2) | 159.6 (9.5) | 27.2 (5.2) | 58.00% | European |
| CHS | 2754 | 166.6 (27.9) | 2833 | 88.3 (10.1) | 2537 | 414.2 (31.3) | N/A | N/A | 72.1 (5.2) | 164.3 (9.2) | 26.2 (4.5) | 1781 (62.9) | European |
| ERF | 2370 | 153.0 (22.6) | 2177 | 97.1 (10.1) | 2332 | 398.5 (28.3) | 2006 | 951.0 (125.8) | 48.0 (14.2) | 167.7 (9.4) | 26.8 (4.6) | 1361 (55.7%) | European |
| FVG | 981 | 156.6 (22.6) | 981 | 94.8 (9.6) | 981 | 399.0 (29.8) | N/A | N/A | 48.5 (21.6) | 168.1 (10.1) | 24.8 (4.5) | 57.29% | European |
| GRAPHIC | 950 | 160 (20) | 942 | 90 (10) | 940 | 410 (20) | N/A | N/A | 52.9 (4.4) | 170.0 (9.0) | 27.5 (4.3) | 509 (50.1%) | European |
| Inter99 | 5907 | 158.3 (22.4) | 5832 | 91.1 (9.9) | 5869 | 403.5 (27.0) | N/A | N/A | 46.1 (7.9) | 172.2 (9.2) | 26.3 (4.6) | 51.10% | European |
| JHS | 2070 | 170.9 (26.2) | 2048 | 92.0 ( 9.9) | 2048 | 410.3 (30.3) | N/A | N/A | 49.9 (12.1) | 169.8 (9.4) | 32.3 (7.8) | 60.56% | African American |
| KORA F3 | 2709 | 158.1 (20.7) | 2599 | 91.9 (9.7) | 2802 | 400.6 (29.4) | N/A | N/A | 27.3 (12.9) | 167.8 (9.4) | 27.6 (4.6) | 51% | European |
| KORA S4 | 3439 | 162.4 (22.2) | 3273 | 91.5 (9.0) | 3542 | 407.5 (27.4) | N/A | N/A | 49.2 (13.9) | 168.3 (9.3) | 27.2 (4.7) | 51% | European |
| Lifelines | 12829 | 157.3 (21.8) | 8817 | 92.6 (10.5) | 8817 | 392.2 (24.8) | 11519 | 906.2 (132.5) | 48.7 (11.5) | 174.4 (9.2) | 26.4 (4.3) | 7744 (58.2) | European |
| NTR | N/A | N/A | N/A | N/A | N/A | N/A | 1482 | 898.1 (125.5) | 27.9 (11.8) | N/A | 23.1 (3.6) | 882 (59.5) | European |
| Prevend | 3301 | 159.1 (21.2) | 3159 | 97.7 (10.6) | 2446 | 395.1 (25.4) | 3159 | 892.3 (132.0) | 49.6 (12.5) | 173.7 (9.1) | 26.1 (4.3) | 1768 (48.5) | European |
| PROSPER | 4237 | 168.2(27.9) | 3976 | 92.9 (10.9) | 4571 | 414.5 (36.3) | 3200 | 888.0 (126.8) | 75.3 (3.4) | 165.2 (9.4) | 26.8 (4.2) | 2720 (51.9) | European |
| RS I | 4455 | 167.0 (24.5) | 3442 | 96.0 (10.5) | 4261 | 397.5 (28.4) | 3456 | 862.5 (124.5) | 68.1 (8.3) | 167.0 (9.3) | 26.3 (3.6) | 61.7 | European |
| RS II | 1631 | 164.7 (22.3) | 1353 | 96.6 (10.7) | 1541 | 401.3 (27.2) | 1354 | 867.6 (120.6) | 64.5 (7.6) | 168.5 (9.2) | 27.3 (4.1) | 55.9 | European |
| RS III | 2872 | 162.9 (21.3) | 2585 | 97.3 (10.5) | 2826 | 401.5 (26.6) | 2522 | 873.0 (122.3) | 56.9 (6.5) | 170.9 (9.6) | 27.6 (4.6) | 57.7 | European |
| TwinsUK | 1807 | 158.0 (22.3) | 1809 | 88.1 (8.4) | 1788 | 403.1 (26.3) | N/A | N/A | 51.2 (13.2) | 163.2 (7.5) | 25.9 (4.7) | 91.5 | European |
| YFS | 1865 | 194.0 (22.7) | 1923 | 84.4 (14.3) | 1841 | 351.7 (26.5) | N/A | N/A | 31.6 (5.0) | 172.2 (9.1) | 25.1 (4.4) | 1320 (54.0) | European |

**Supplementary Table 2: Genotype characteristics**

| Cohort name | Genotyping array | Sample size | Call rate | Other exclusion criteria | Sample size in analysis | MAF | Call rate | HWE P-value | Other exclusion criteria | n SNPs before imputation | Reference panel | Quality control | Imputation | GWAS |
| --- | --- | --- | --- | --- | --- | --- | --- | --- | --- | --- | --- | --- | --- | --- |
| AGES | Hu370CNV, Illumina | 3660 | 97% | Mismatch previous genotypes, mismatch predicted sex, poor Illumina quality | 3219 | 0.01 | 97% | 1 x 10-6 | SNPs not in Hapmap, remove G/C A/T SNPs | 324603 | 1000G phase 1 v3 | PLINK | Mach/Minimac | R |
| ARIC | Affymetrix 6.0 | 9747 | 95% | 1st degree relatives, genetic outliers, not-matching existing genotype data | 8040 | 0.005 | 95% | 1 x 10-5 | N/A | 682749 autosomal | 1000G phase 1 v3 | PLINK | IMPUTE2 | FAST |
| BRIGHT | Affymetrix GeneChip 500k array | 2001 | 97% | Heterozygosity Outliers, Non European ancestry, duplicates, 1st or 2nd degree relatives, sex mismatch | 1,948 | >0 | N/A | 1 x 10-7 | N/A | 446472 | 1000G phase 1 v Dec 2013 | PLINK | Minimac | R |
| CHS | Illumina 370CNV and ITMAT-Broad-CARe (IBC) Illumina iSELECT chip | 3268 | 95% | Disconcordance with prior genotyping, sex mismatch | 3,268 | >0 | 97% | 1 x 10-5 | Errors in duplicate samples, Mendelian inconstancies in CEPH trios, hetero-zygote frequency of 0 | 359592 | 1000G phase 1 v3 | R and BeadStudio | Minimac | R |
| ERF | Illumina 318/370 K, Affymetrix 250 K, and Illumina 6 K | 2492 | 95% | No ECG or missing covariate or exclusion information. | 2442 | 0.01 | 98% | 1 x 10-6 | Mendelian error | 678524 | GoNL v4 | BeadStudio, R | miniMACH | GenABEL/ProbABEL |
| GRAPHIC | Illumina HumanOmniExpress-12v1 | 1017 | 95% | N/A | N/A | N/A | N/A | N/A | N/A | N/A | 1000 Genomes March 2012 Version 3 | N/A | IMPUTE2 | SNPTEST v2.5 |
| INGI-Carlantino | Illumina 370K | 1024 | 95% | NA | 326 | 0.01 | 97% | 1 x 10-5 | N/A | 310162 | 1000G phase 1 v3 | GenABEL | IMPUTE2 | GenABEL |
| INGI-FVG | Illumina 370K | 1794 | 95% | NA | 981 | 0.01 | 97% | 1 x 10-5 | N/A | 337266 | 1000G phase 1 v3 | GenABEL | IMPUTE2 | GenABEL |
| Inter99 | MetaboChip | 5951 | 95% |  | 5951 |  | 95% | 1 x 10-5 | N/A | N/A | N/A | PLINK, R | NA | PLINK |
| JHS | Illumina HumanExome BeadChip array | 2986 | 95% | Ethnic outliers; individuals with high inbreeding coefficient defined as F >0.2 or < -0.2; Sex mismatch; duplicated indivi-duals identified as IBD sharing π hat >0.9; individuals overlapped with ARIC cohort; poor concordance; indi-vidual with missing genotype > 5% | 2125 | N/A | 95% | 1 x 10-6 | N/A | N/A | 1000G phase 1 v3 | R, PLINK and EIGENSOFT | MaCH | Linear mixed-effect model using the lmekin function of the R package kinship. |
| KORA F3 | Illumina Omni 2.5 + Illumina Omni Express | 4086 | 97% | excess heterozygosity, population outlier, sex check, phenotype availability | 2709 | 0.01 | 98% | 5 x 10-6 | availability in good quality on both chips | 600,641 | 1000genomes phase 1 all | R | SHAPEIT v2 + IMPUTE v2.3.0 | R |
| KORA S4 | Affymetrix Axiom | 3788 | 97% |  | 3788 | 0.01 | 98% | 5 x 10-6 |  | 523,260 | 1000genomes phase 1 all | R | SHAPEIT v2 + IMPUTE v2.3.0 | R |
| Lifelines | Illumina CytoSNP12 v2 | 13,302 | 95% | IBS (IBS>0.125) | 13,302 | 0.01 | 95% | 1 x 10-5 | Sex mismatch, outliers based on PCA analysis. | 257581 | GoNL v4 | MOLGENIS | Impute2 | SNPtest v2 |
| NTR | Affymetrix, 5.0,; Illumina 370; Illumina 660, Illumina Omni Express 1 M,Affymetrix 6.0 | 1482 | 90% | non-Dutch ancestry | 1482 | 0.01 | 95% | 1 x 10-5 | imputation quality cutoff R2 < 0.30. | N/A | GoNL v4 | PLINK, R | MaCH (version 1.0.18), Minimac (version 2012.10.9 beta) | Plink |
| Prevend | Illumina CytoSNP12 v2 | 3,648 | 95% | IBS (IBS>0.125) | 3,648 | 0.01 | 95% | 1 x 10-5 | Sex mismatch, outliers based on PCA analysis. | 232571 | GoNL v4 | MOLGENIS | Impute2 | SNPtest v2 |
| PROSPER | Illumina 660K Quad beadchip | 5786 | 98% | family relatedness, gender mismatch, ancestry | 5244 | 0.01 | 97.5% | 1 x 10-6 | N/A | 557192 | GoNL v4 | BeadStudio | IMPUTE | SNPTEST |
| RS I | Illumina HumanHap 550K V.3 ADHumanHap 550 V.3 DUO | 7893 | 97.5% | Duplicate samples, sex mismatch | 6291 | 0.01 | 98% | 1 x 10-6 | Sample call rate < 98%, Missing DNA, Gender mismatch, Excess autosomal heterozigocity, Duplicates or family relations IBS>97%, Ethnic outliers (IBS distances > 4SD, Missing traits | 512,849 | GoNL v4 | PLINK | Impute2 2.3.0 | GRIMP |
| RS II | Illumina HumanHap 550K V.3 ADHumanHap 550 V.3 DUO | 3011 | 97.5% | Duplicate samples, sex mismatch | 2160 | 0.01 | 98% | 1 x 10-6 |  | 466,389 | GoNL v4 | PLINK | Impute2 2.3.0 | GRIMP |
| RS III | Illumina HumanHap 610 quad | 3932 | 97.5% | Duplicate samples, sex mismatch | 3045 | 0.01 | 98% | 1 x 10-6 |  | 514,073 | GoNL v4 | PLINK | Impute2 2.3.0 | GRIMP |
| TwinsUK | Illumina HumanHap300, Illumina Human610 arrays | 2575 | N/A | exclusions basex population stratification, heterozygosity, zygosity and sex checks | Up to 1809 | 0.01 | N/A | N/A | N/A | N/A | 1000 Genomes phase 1 v3 + UK10K Whole Genome Sequence (REL-2012-06-02, v2, N=3,781) | PLINK, R | SHAPEIT version 2 , IMPUTE version 2.2.0 | GEMMA version 0.93 SNPTEST version 2.4.1 |
| YFS | Illumina Human670-QuadCustom | 2556 | 95% | gender mismatch, excess heterozygosity, cryptic relatedness | 2443 | 0.01 | 95% | 1 x 10-6 | N/A | 546674 | 1000 Genomes phase I integrated variant set release (v3) | Sanger genotyping pipe-line qc | IMPUTE v2.2.2 | SNPTEST v2.4.1 |

**Supplementary Table 3: Exclusion criteria**

| **Exclusion criteria** | **PR** | **QT** | **QRS** | **RR** |
| --- | --- | --- | --- | --- |
| Pacemaker | X | X | X | X |
| Atrial fibrillation on baseline ECG | X | X | X | X |
| History of Myocardial Infarction or Heart failure | X |  | X | X |
| Pregnancy | X | X |  |  |
| Wolff-Parkinson-White | X |  | X |  |
| Class I and class III blocking medication | X |  | X |  |
| Second or third degree heart block | X |  |  | X |
| Digoxin | X |  |  | X |
| QRS duration > 120ms or left or right bundle branch block and intraventricular conduction delay |  | X | X |  |
| Extreme PR values (<=80ms or >=320 ms) | X |  |  |  |
| Use of QT-shortening or QT-prolonging drugs |  | X |  |  |
| Coded as incomplete LBTB, RBTB |  |  | X |  |
| QRS axis smaller than -30 or larger than +90 (or left anterior hemiblock and left posterior hemiblock) |  |  | X |  |
| Heart rate < 50 BPM or > 100 BPM |  |  |  | X |
| Use of beta blockers or non-dihydropyridin calcium-antagonists date of ECG |  |  |  | X |

**Supplementary Table 4: Association results for RR, PR, QRS, and QT.** Independent genome-wide significant SNPs are listed.

| **Locus** | **SNP** | | **Chr** | | **Pos (hg19)** | **Coded allele** | **Non-coded allele** | **Coded allele frequency** | **Beta** | | **SE** | **P-value** | | | **Conditional**  **P-value** | **Closest gene** |
| --- | --- | --- | --- | --- | --- | --- | --- | --- | --- | --- | --- | --- | --- | --- | --- | --- |
| RR-1 | rs2785638 | | 1 | | 208093719 | G | A | 0.474 | -6.429 | | 1.094 | 4.19E-09 | | |  | CD34 |
| RR-2 | rs6911599 | | 6 | | 119009913 | G | A | 0.495 | -6.727 | | 1.069 | 3.14E-10 | | |  | CEP85L |
| RR-3-1 | rs9385199 | | 6 | | 121837314 | G | A | 0.584 | 6.672 | | 1.130 | 3.49E-09 | | | 2.23E-07 | GJA1 |
| RR-3-2 | rs9372667 | | 6 | | 122145892 | G | T | 0.106 | -12.924 | | 1.739 | 1.09E-13 | | |  | GJA1 |
| RR-4 | rs3757868 | | 7 | | 100482720 | A | G | 0.191 | -8.956 | | 1.375 | 7.23E-11 | | |  | SRRT |
| RR-5 | rs9888363 | | 12 | | 33529288 | C | T | 0.614 | 6.601 | | 1.134 | 5.85E-09 | | |  | SYT10 |
| RR-6 | rs432256 | | 14 | | 23870752 | A | G | 0.250 | -8.122 | | 1.245 | 6.89E-11 | | |  | MYH6 |
| RR-7 | rs6069234 | | 20 | | 36843992 | G | A | 0.481 | 6.553 | | 1.138 | 8.58E-09 | | |  | KIAA1755 |
|  |  | |  | |  |  |  |  |  | |  |  | | |  |  |
| **Locus** | **SNP** | | **Chr** | | **Pos (hg19)** | **Coded allele** | **Non-coded allele** | **Coded allele frequency** | **Beta** | | **SE** | **P-value** | | | **Conditional**  **P-value** | **Closest gene** |
| PR-1 | rs2503715 | | 1 | | 2144107 | G | A | 0.878 | 2.221 | | 0.343 | 9.93E-11 | | |  | AL590822.1 |
| PR-2-1 | rs12145374 | | 1 | | 112480536 | C | A | 0.203 | -1.419 | | 0.238 | 2.40E-09 | | | 1.03E-06 | KCND3 |
| PR-2-2 | rs75013985 | | 1 | | 112530430 | G | A | 0.033 | -4.090 | | 0.554 | 1.48E-13 | | |  | KCND3 |
| PR-3 | rs6724747 | | 2 | | 66749828 | A | G | 0.346 | 1.379 | | 0.193 | 7.90E-13 | | |  | MEIS1 |
| PR-4-1 | rs7623452 | | 3 | | 37616229 | A | G | 0.521 | 1.061 | | 0.185 | 9.91E-09 | | | 5.19E-07 | ITGA9 |
| PR-4-2 | rs3828441 | | 3 | | 38356737 | A | G | 0.225 | -1.384 | | 0.251 | 3.59E-08 | | | 3.21E-06 | SLC22A14 |
| PR-4-3 | rs73070938 | | 3 | | 38606159 | A | C | 0.108 | 2.109 | | 0.328 | 1.34E-10 | | | 2.94E-11 | SCN5A |
| PR-4-4 | rs62241190 | | 3 | | 38607468 | G | A | 0.035 | 5.926 | | 0.617 | 7.26E-22 | | | 5.02E-13 | SCN5A |
| PR-4-5 | rs3922843 | | 3 | | 38624343 | G | A | 0.748 | 2.622 | | 0.219 | 3.53E-33 | | | 4.00E-14 | SCN5A |
| PR-4-6 | rs39351484 | | 3 | | 38638202 | G | C | 0.665 | -1.733 | | 0.198 | 2.05E-18 | | | 3.26E-19 | SCN5A |
| PR-4-7 | rs9832895 | | 3 | | 38661533 | C | T | 0.524 | 1.210 | | 0.209 | 7.46E-09 | | | 4.79E-18 | SCN5A |
| PR-4-8 | rs7373065 | | 3 | | 38710315 | C | T | 0.978 | 8.200 | | 0.726 | 1.45E-29 | | | 6.12E-17 | SCN5A |
| PR-4-9 | rs6801957 | | 3 | | 38767315 | C | T | 0.584 | -4.062 | | 0.185 | 4.91E-107 | | |  | SCN10A |
| PR-4-10 | rs9828912 | | 3 | | 38837009 | A | T | 0.083 | 2.714 | | 0.471 | 8.06E-09 | | | 1.47E-06 | SCN10A |
| PR-4-11 | rs62244116 | | 3 | | 38845547 | C | A | 0.052 | 4.401 | | 0.526 | 5.74E-17 | | | 4.07E-07 | SCN10A |
| PR-5 | rs4461368 | | 3 | | 73599305 | T | C | 0.626 | 1.102 | | 0.195 | 1.54E-08 | | |  | PDZRN3 |
| PR-6 | rs13111293 | | 4 | | 86682968 | T | C | 0.703 | 1.881 | | 0.200 | 5.79E-21 | | |  | ARHGAP24 |
| PR-7 | rs17287745 | | 5 | | 142655015 | G | A | 0.425 | 1.011 | | 0.185 | 4.24E-08 | | |  | NR3C1 |
| PR-8 | rs29797 | | 5 | | 172574176 | C | G | 0.573 | 1.283 | | 0.186 | 4.85E-12 | | |  | BNIP1 |
| PR-9 | rs74640693 | | 6 | | 118684824 | T | A | 0.049 | 2.376 | | 0.428 | 2.88E-08 | | |  | SLC35F1 |
| PR-10 | rs11763856 | | 7 | | 35545787 | T | C | 0.035 | 3.055 | | 0.539 | 1.43E-08 | | |  | HERPUD2 |
| PR-11 | rs11773845 | | 7 | | 116191301 | A | C | 0.573 | -2.012 | | 0.184 | 9.18E-28 | | |  | CAV1 |
| PR-12 | rs10842383 | | 12 | | 24771967 | T | C | 0.144 | -1.882 | | 0.262 | 6.29E-13 | | |  | BCAT1 |
| PR-13-1 | rs6489973 | | 12 | | 115156523 | G | A | 0.412 | -1.157 | | 0.186 | 4.92E-10 | | |  | TBX3 |
| PR-13-2 | rs7301677 | | 12 | | 115381147 | T | C | 0.272 | 1.275 | | 0.207 | 6.78E-10 | | | 9.27E-10 | TBX3 |
| PR-14 | rs11840168 | | 13 | | 22118302 | G | A | 0.413 | -1.280 | | 0.187 | 6.88E-12 | | |  | EFHA1 |
| PR-15 | rs9534461 | | 13 | | 47241834 | A | T | 0.642 | -1.229 | | 0.190 | 1.08E-10 | | |  | LRCH1 |
| PR-16 | rs8019721 | | 14 | | 71834210 | A | G | 0.261 | 1.192 | | 0.209 | 1.13E-08 | | |  | AC004817.1 |
| PR-17 | rs35712872 | | 17 | | 12635871 | G | A | 0.233 | 1.408 | | 0.245 | 9.08E-09 | | |  | MYOCD |
|  |  | |  | |  |  |  |  |  | |  |  | | |  |  |
| **Locus** | **SNP** | | **Chr** | | **Pos (hg19)** | **Coded allele** | **Non-coded allele** | **Coded allele frequency** | **Beta** | | **SE** | **P-value** | | | **Conditional**  **P-value** | **Closest gene** |
| QT-1 | rs11121483 | | 1 | | 6263792 | G | A | 0.392 | 1.508 | | 0.219 | 5.49E-12 | | |  | RNF207 |
| QT-2 | rs6588213 | | 1 | | 67107894 | T | C | 0.126 | 1.596 | | 0.282 | 1.53E-08 | | |  | SGIP1 |
| QT-3-1 | rs142804708 | | 1 | | 162012135 | T | C | 0.045 | -5.351 | | 0.870 | 7.59E-10 | | | 1.52E-07 | OLFML2B |
| QT-3-2 | rs2010491 | | 1 | | 162021000 | G | A | 0.233 | 4.332 | | 0.216 | 2.01E-89 | | |  | NOS1AP |
| QT-3-3 |  | | 1 | | 162030285 | A | G | 0.142 | 4.671 | | 0.329 | 1.17E-45 | | | 9.44E-10 | NOS1AP |
| QT-3-4 | rs10918592 | | 1 | | 162053449 | T | C | 0.021 | 7.231 | | 0.851 | 1.89E-17 | | | 1.90E-10 | NOS1AP |
| QT-3-5 | rs59852339 | | 1 | | 162112966 | C | T | 0.109 | 3.206 | | 0.300 | 1.16E-26 | | | 2.04E-10 | NOS1AP |
| QT-3-6 | rs12567315 | | 1 | | 162166646 | A | G | 0.196 | 3.331 | | 0.228 | 2.91E-48 | | | 2.59E-13 | NOS1AP |
| QT-3-7 | rs115263373 | | 1 | | 162169913 | G | T | 0.056 | -2.356 | | 0.402 | 4.41E-09 | | | 5.28E-11 | NOS1AP |
| QT-4-1 | rs77915002 | | 1 | | 168686870 | G | A | 0.056 | 2.169 | | 0.393 | 3.30E-08 | | | 5.68E-10 | DPT |
| QT-4-2 | rs1200118 | | 1 | | 169064630 | G | A | 0.478 | -1.369 | | 0.237 | 8.10E-09 | | | 1.77E-11 | ATP1B1 |
| QT-4-3 | rs12035622 | | 1 | | 169102340 | A | T | 0.117 | -2.287 | | 0.282 | 5.54E-16 | | |  | NME7 |
| QT-5 | rs11097788 | | 4 | | 103407428 | G | A | 0.561 | 1.048 | | 0.186 | 1.81E-08 | | |  | NFKB1 |
| QT-6-1 | rs11153730 | | 6 | | 118667522 | C | T | 0.499 | 2.249 | | 0.181 | 1.80E-35 | | |  | SLC35F1 |
| QT-6-2 | rs12206973 | | 6 | | 118711303 | C | G | 0.050 | -3.450 | | 0.423 | 3.38E-16 | | | 1.63E-10 | CEP85L |
| QT-6-3 | rs6911599 | | 6 | | 119009913 | G | A | 0.497 | -2.128 | | 0.182 | 1.60E-31 | | | 6.49E-08 | CEP85L |
| QT-7-1 | rs2072412 | | 7 | | 150647970 | G | C | 0.244 | -1.885 | | 0.225 | 4.61E-17 | | | 4.31E-08 | KCNH2 |
| QT-7-2 | rs12668582 | | 7 | | 150657201 | C | A | 0.322 | 1.712 | | 0.198 | 4.64E-18 | | |  | KCNH2 |
| QT-8-1 | rs800340 | | 11 | | 2458393 | A | T | 0.980 | 7.264 | | 1.284 | 1.54E-08 | | | 5.04E-06 | LSP1 |
| QT-8-2 | rs800338 | | 11 | | 2473456 | A | G | 0.844 | 2.287 | | 0.268 | 1.33E-17 | | | 1.24E-19 | KCNQ1 |
| QT-8-3 | rs12271931 | | 11 | | 2478519 | A | G | 0.869 | 3.035 | | 0.337 | 7.40E-20 | | | 1.02E-13 | KCNQ1 |
| QT-8-4 | rs2074238 | | 11 | | 2484803 | C | T | 0.920 | 5.405 | | 0.494 | 7.34E-28 | | |  | KCNQ1 |
| QT-8-5 | rs12280952 | | 11 | | 2488873 | A | G | 0.164 | 2.498 | | 0.250 | 1.56E-23 | | | 1.01E-22 | KCNQ1 |
| QT-9 | rs174546 | | 11 | | 61569830 | T | C | 0.334 | -1.238 | | 0.204 | 1.35E-09 | | |  | FADS1 |
| QT-10 | rs28637922 | | 12 | | 110819139 | T | G | 0.261 | 1.173 | | 0.213 | 3.64E-08 | | |  | ANAPC7 |
| QT-11 | rs11643990 | | 16 | | 11694062 | A | G | 0.333 | -1.355 | | 0.203 | 2.61E-11 | | |  | LITAF |
| QT-12 | rs950843 | | 16 | | 58620885 | T | C | 0.758 | 1.977 | | 0.212 | 9.57E-21 | | |  | CNOT1 |
| QT-13 | rs2074518 | | 17 | | 33324382 | T | C | 0.462 | -1.330 | | 0.183 | 3.43E-13 | | |  | LIG3 |
| QT-14 | rs4793397 | | 17 | | 68520389 | T | C | 0.464 | -1.178 | | 0.187 | 2.87E-10 | | |  | KCNJ2 |
| QT-15 | rs1805128 | | 21 | | 35821680 | T | C | 0.018 | 7.409 | | 0.939 | 2.91E-15 | | |  | KCNE1 |
|  |  | |  | |  |  |  |  |  | |  |  | | |  |  |
| **Locus** | **SNP** | | **Chr** | | **Pos (hg19)** | **Coded allele** | **Non-coded allele** | **Coded allele frequency** | **Beta** | | **SE** | **P-value** | | | **Conditional**  **P-value** | **Closest gene** |
| QRS-1 | rs149288352 | | 1 | | 51349663 | A | G | 0.025 | -1.515 | | 0.275 | 3.67E-08 | | |  | FAF1 |
| QRS-2 | rs6587924 | | 1 | | 61895257 | A | C | 0.508 | -0.822 | | 0.086 | 1.89E-21 | | |  | NFIA |
| QRS-3-1 | rs7429945 | | 3 | | 38591689 | C | T | 0.345 | 0.956 | | 0.092 | 2.19E-25 | | | 1.78E-15 | SCN5A |
| QRS-3-2 | rs62241190 | | 3 | | 38607468 | G | A | 0.034 | 2.524 | | 0.293 | 6.32E-18 | | | 1.99E-10 | SCN5A |
| QRS-3-3 | rs3922843 | | 3 | | 38624343 | G | A | 0.747 | 0.770 | | 0.104 | 1.08E-13 | | | 1.68E-11 | SCN5A |
| QRS-3-4 | rs9851710 | | 3 | | 38719901 | A | C | 0.654 | 1.011 | | 0.093 | 2.53E-27 | | |  | SCN10A |
| QRS-3-5 | rs4076737 | | 3 | | 38764782 | T | G | 0.549 | -0.895 | | 0.090 | 2.68E-23 | | | 3.47E-16 | SCN10A |
| QRS-4 | rs10076436 | | 5 | | 153871841 | G | C | 0.358 | -0.809 | | 0.092 | 9.68E-19 | | |  | HAND1 |
| QRS-5 | rs3176326 | | 6 | | 36647289 | A | G | 0.202 | 1.177 | | 0.114 | 4.49E-25 | | |  | CDKN1A |
| QRS-6-1 | rs3951016 | | 6 | | 118559658 | A | T | 0.459 | -1.004 | | 0.088 | 2.52E-30 | | |  | SLC35F1 |
| QRS-6-2 | rs10457327 | | 6 | | 118692152 | C | G | 0.053 | -1.281 | | 0.196 | 5.80E-11 | | | 1.35E-06 | SLC35F1 |
| QRS-7-1 | rs13232979 | | 7 | | 35310657 | A | T | 0.117 | 0.841 | | 0.139 | 1.24E-09 | | |  | TBX20 |
| QRS-7-2 | rs11764098 | | 7 | | 35505710 | G | C | 0.030 | 1.711 | | 0.287 | 2.50E-09 | | | 9.39E-07 | HERPUD2 |
| QRS-8 | rs6585178 | | 10 | | 114479277 | G | A | 0.239 | 0.714 | | 0.103 | 4.67E-12 | | |  | VTI1A |
| QRS-9 | rs28637922 | | 12 | | 110819139 | T | G | 0.259 | 0.565 | | 0.102 | 3.02E-08 | | |  | ANAPC7 |
| QRS-10-1 | rs1248048 | 12 | | 114856951 | | G | C | 0.724 | -0.570 | 0.098 | | | 5.38E-09 | 4.32E-08 | | TBX5 |
| QRS-10-2 | rs7487237 | 12 | | 115353703 | | G | A | 0.719 | 0.580 | 0.099 | | | 4.54E-09 |  | | TBX3 |
| QRS-11 | rs728926 | 13 | | 74513122 | | T | C | 0.375 | -0.581 | 0.094 | | | 5.76E-10 |  | | KLF12 |
| QRS-12 | rs34991781 | 14 | | 71921576 | | A | G | 0.264 | -0.810 | 0.099 | | | 2.25E-16 |  | | SIPA1L1 |
| QRS-13 | rs11661654 | 18 | | 42441443 | | T | C | 0.415 | 0.598 | 0.089 | | | 1.95E-11 |  | | SETBP1 |

**Supplementary Table 5: Nodes that are significantly enriched for genes that are mapped to index SNPs.** In total, 100 independent locus-trait associations were merged and mapped to genes using GREAT. SNPs were mapped to a gene is it was in a *cis*-regulatory region of that gene. Significant nodes are listed for GO terms, human phenotypes, and disease ontologies.

Supplementary Table 5a: GO molecular function

| **# Term Name** | **Binom Rank** | **Binom Raw P-Value** | **Binom FDR Q-Val** | **Binom Fold Enrichment** | **Binom Observed Region Hits** | **Binom Region Set Coverage** |
| --- | --- | --- | --- | --- | --- | --- |
| nitric-oxide synthase binding | 1 | 5.34E-60 | 1.97E-56 | 234.5939 | 29 | 0.29 |
| scaffold protein binding | 4 | 1.08E-38 | 1.00E-35 | 70.38353 | 25 | 0.25 |
| voltage-gated sodium channel activity | 5 | 1.21E-38 | 8.91E-36 | 138.1109 | 21 | 0.21 |
| sodium channel activity | 6 | 2.07E-38 | 1.27E-35 | 79.55073 | 24 | 0.24 |
| sodium ion transmembrane transporter activity | 9 | 4.16E-28 | 1.71E-25 | 23.54955 | 26 | 0.26 |
| voltage-gated cation channel activity | 11 | 2.71E-27 | 9.09E-25 | 14.49064 | 31 | 0.31 |
| voltage-gated ion channel activity | 12 | 3.60E-25 | 1.11E-22 | 12.27158 | 31 | 0.31 |
| monovalent inorganic cation transmembrane transporter activity | 13 | 4.95E-23 | 1.41E-20 | 9.234968 | 33 | 0.33 |
| metal ion transmembrane transporter activity | 14 | 1.73E-22 | 4.55E-20 | 7.635562 | 36 | 0.36 |
| enzyme binding | 15 | 4.68E-22 | 1.15E-19 | 4.406918 | 51 | 0.51 |
| cation channel activity | 16 | 1.64E-20 | 3.79E-18 | 8.465701 | 31 | 0.31 |
| inorganic cation transmembrane transporter activity | 17 | 1.69E-20 | 3.66E-18 | 6.637147 | 36 | 0.36 |
| cation transmembrane transporter activity | 19 | 4.25E-19 | 8.25E-17 | 5.771739 | 37 | 0.37 |
| ion gated channel activity | 20 | 9.51E-19 | 1.75E-16 | 7.333553 | 31 | 0.31 |
| channel activity | 21 | 1.55E-18 | 2.73E-16 | 6.548074 | 33 | 0.33 |
| substrate-specific transmembrane transporter activity | 23 | 1.08E-17 | 1.73E-15 | 4.715709 | 40 | 0.4 |
| ion transmembrane transporter activity | 24 | 1.78E-17 | 2.74E-15 | 4.801418 | 39 | 0.39 |
| ion channel activity | 26 | 3.56E-17 | 5.05E-15 | 6.439358 | 31 | 0.31 |
| substrate-specific channel activity | 27 | 4.66E-17 | 6.37E-15 | 6.376701 | 31 | 0.31 |
| transmembrane transporter activity | 28 | 1.57E-16 | 2.07E-14 | 4.361665 | 40 | 0.4 |
| substrate-specific transporter activity | 29 | 2.08E-16 | 2.64E-14 | 4.326363 | 40 | 0.4 |
| transporter activity | 30 | 9.13E-15 | 1.12E-12 | 3.76182 | 41 | 0.41 |
| voltage-gated potassium channel activity involved in ventricular cardiac muscle cell action potential repolarization | 32 | 1.91E-14 | 2.20E-12 | 182.3488 | 7 | 0.07 |
| voltage-gated potassium channel activity involved in cardiac muscle cell action potential repolarization | 33 | 1.78E-13 | 1.98E-11 | 79.65021 | 8 | 0.08 |
| potassium ion transmembrane transporter activity | 47 | 7.52E-07 | 5.90E-05 | 6.066083 | 12 | 0.12 |
| voltage-gated potassium channel activity | 48 | 1.50E-06 | 1.15E-4 | 7.190786 | 10 | 0.1 |
| transcription factor binding | 50 | 6.12E-06 | 4.52E-4 | 3.347959 | 18 | 0.18 |
| potassium channel activity | 55 | 1.68E-05 | 0.0011 | 5.44302 | 10 | 0.1 |
| transcription regulatory region sequence-specific DNA binding | 73 | 2.90E-4 | 0.0146 | 3.845833 | 10 | 0.1 |

Supplementary Table 5b: GO biological process

| **# Term Name** | **Binom Rank** | **Binom Raw P-Value** | **Binom FDR Q-Val** | **Binom Fold Enrichment** | **Binom Observed Region Hits** | **Binom Region Set Coverage** |
| --- | --- | --- | --- | --- | --- | --- |
| regulation of cardiac muscle cell membrane potential | 1 | 4.33E-61 | 4.52E-57 | 64.66527 | 40 | 0.4 |
| cardiac conduction | 2 | 4.09E-58 | 2.13E-54 | 65.51545 | 38 | 0.38 |
| membrane repolarization | 3 | 5.16E-55 | 1.80E-51 | 118.3191 | 31 | 0.31 |
| regulation of ventricular cardiac muscle cell membrane repolarization | 4 | 7.06E-55 | 1.84E-51 | 182.6363 | 28 | 0.28 |
| regulation of cardiac muscle cell contraction | 5 | 7.07E-54 | 1.48E-50 | 61.16414 | 36 | 0.36 |
| membrane repolarization involved in regulation of cardiac muscle cell action potential | 6 | 4.06E-52 | 7.06E-49 | 125.1537 | 29 | 0.29 |
| regulation of atrial cardiac muscle cell membrane repolarization | 7 | 3.64E-51 | 5.42E-48 | 336.9142 | 23 | 0.23 |
| regulation of atrial cardiac muscle cell membrane depolarization | 8 | 2.53E-50 | 3.31E-47 | 309.5837 | 23 | 0.23 |
| regulation of heart rate | 9 | 1.10E-49 | 1.27E-46 | 35.97524 | 39 | 0.39 |
| regulation of heart rate by cardiac conduction | 10 | 1.21E-49 | 1.26E-46 | 118.505 | 28 | 0.28 |
| regulation of cardiac muscle contraction | 11 | 4.50E-49 | 4.27E-46 | 34.6758 | 39 | 0.39 |
| bundle of His cell to Purkinje myocyte communication | 12 | 5.86E-49 | 5.09E-46 | 337.0679 | 22 | 0.22 |
| regulation of striated muscle contraction | 13 | 1.26E-48 | 1.01E-45 | 33.75919 | 39 | 0.39 |
| regulation of membrane repolarization | 14 | 2.76E-48 | 2.06E-45 | 92.13828 | 29 | 0.29 |
| regulation of ventricular cardiac muscle cell action potential | 15 | 8.54E-48 | 5.94E-45 | 117.8716 | 27 | 0.27 |
| regulation of cardiac muscle cell action potential | 16 | 3.92E-47 | 2.56E-44 | 58.58841 | 32 | 0.32 |
| regulation of cardiac muscle cell action potential involved in contraction | 17 | 6.50E-47 | 3.99E-44 | 72.70491 | 30 | 0.3 |
| heart process | 18 | 1.16E-46 | 6.72E-44 | 50.94095 | 33 | 0.33 |
| AV node cell to bundle of His cell communication | 19 | 2.61E-46 | 1.44E-43 | 321.0421 | 21 | 0.21 |
| membrane depolarization involved in regulation of action potential | 20 | 1.15E-45 | 6.00E-43 | 114.2408 | 26 | 0.26 |
| cell-cell signaling involved in cardiac conduction | 21 | 7.95E-45 | 3.95E-42 | 105.9987 | 26 | 0.26 |
| regulation of heart contraction | 22 | 4.67E-44 | 2.21E-41 | 19.65178 | 43 | 0.43 |
| regulation of muscle contraction | 23 | 8.90E-44 | 4.04E-41 | 21.96013 | 41 | 0.41 |
| cell communication involved in cardiac conduction | 24 | 3.11E-43 | 1.35E-40 | 69.666 | 28 | 0.28 |
| membrane depolarization involved in regulation of cardiac muscle cell action potential | 25 | 6.75E-42 | 2.82E-39 | 111.4422 | 24 | 0.24 |
| regulation of ventricular cardiac muscle cell membrane depolarization | 26 | 8.42E-42 | 3.38E-39 | 195.548 | 21 | 0.21 |
| regulation of muscle system process | 30 | 2.70E-40 | 9.38E-38 | 17.95286 | 41 | 0.41 |
| muscle system process | 34 | 5.93E-38 | 1.82E-35 | 14.79662 | 42 | 0.42 |
| cardiac muscle contraction | 36 | 8.13E-38 | 2.36E-35 | 56.64215 | 26 | 0.26 |
| heart contraction | 37 | 8.15E-38 | 2.30E-35 | 49.91578 | 27 | 0.27 |
| regulation of membrane depolarization | 38 | 8.19E-38 | 2.25E-35 | 87.9581 | 23 | 0.23 |
| sodium ion transmembrane transport | 39 | 5.91E-36 | 1.58E-33 | 62.69731 | 24 | 0.24 |
| striated muscle contraction | 41 | 1.96E-35 | 4.98E-33 | 40.61466 | 27 | 0.27 |
| regulation of metal ion transport | 42 | 3.77E-34 | 9.37E-32 | 14.85358 | 38 | 0.38 |
| regulation of membrane potential | 43 | 4.27E-34 | 1.04E-31 | 10.75013 | 44 | 0.44 |
| positive regulation of sodium ion transport | 44 | 4.53E-32 | 1.07E-29 | 66.7804 | 21 | 0.21 |
| blood circulation | 45 | 3.69E-31 | 8.56E-29 | 11.63122 | 39 | 0.39 |
| cardiac ventricle development | 46 | 4.28E-31 | 9.71E-29 | 18.0096 | 32 | 0.32 |
| circulatory system process | 47 | 4.87E-31 | 1.08E-28 | 11.54417 | 39 | 0.39 |
| positive regulation of ion transport | 48 | 6.83E-31 | 1.49E-28 | 20.76383 | 30 | 0.3 |
| regulation of ion homeostasis | 49 | 2.87E-30 | 6.12E-28 | 18.24977 | 31 | 0.31 |
| regulation of ion transmembrane transport | 50 | 7.77E-30 | 1.62E-27 | 10.18149 | 40 | 0.4 |
| regulation of action potential | 51 | 9.88E-30 | 2.02E-27 | 15.16162 | 33 | 0.33 |
| membrane depolarization | 52 | 1.26E-29 | 2.52E-27 | 24.46696 | 27 | 0.27 |
| regulation of transmembrane transport | 53 | 1.37E-29 | 2.70E-27 | 10.02939 | 40 | 0.4 |
| regulation of actin filament-based process | 54 | 4.49E-29 | 8.68E-27 | 11.99772 | 36 | 0.36 |
| cardiac chamber development | 55 | 1.11E-28 | 2.10E-26 | 15.02651 | 32 | 0.32 |
| regulation of system process | 56 | 3.40E-28 | 6.34E-26 | 6.889042 | 47 | 0.47 |
| metal ion transport | 57 | 6.53E-28 | 1.20E-25 | 7.313961 | 45 | 0.45 |
| muscle contraction | 58 | 1.14E-27 | 2.06E-25 | 13.91984 | 32 | 0.32 |
| regulation of ion transport | 59 | 2.70E-27 | 4.77E-25 | 7.647608 | 43 | 0.43 |
| sodium ion transport | 61 | 2.48E-26 | 4.25E-24 | 20.01457 | 26 | 0.26 |
| cation transport | 63 | 1.15E-25 | 1.90E-23 | 6.226637 | 46 | 0.46 |
| odontogenesis of dentin-containing tooth | 66 | 1.12E-24 | 1.77E-22 | 20.75163 | 24 | 0.24 |
| heart development | 67 | 6.48E-24 | 1.01E-21 | 6.77108 | 41 | 0.41 |
| cardiovascular system development | 68 | 7.86E-24 | 1.21E-21 | 4.825322 | 51 | 0.51 |
| monovalent inorganic cation transport | 69 | 1.05E-23 | 1.59E-21 | 9.714724 | 33 | 0.33 |
| regulation of homeostatic process | 72 | 8.13E-23 | 1.18E-20 | 9.086706 | 33 | 0.33 |
| ion transmembrane transport | 73 | 1.15E-22 | 1.65E-20 | 7.385485 | 37 | 0.37 |
| odontogenesis | 74 | 1.40E-21 | 1.97E-19 | 15.22058 | 24 | 0.24 |
| regulation of cellular component movement | 75 | 3.15E-21 | 4.39E-19 | 5.72716 | 41 | 0.41 |
| ion transport | 76 | 7.87E-21 | 1.08E-18 | 4.48278 | 48 | 0.48 |
| regulation of multicellular organismal process | 78 | 4.02E-19 | 5.38E-17 | 2.781644 | 66 | 0.66 |
| regulation of transport | 79 | 1.51E-18 | 1.99E-16 | 3.668435 | 51 | 0.51 |
| transmembrane transport | 81 | 1.50E-17 | 1.93E-15 | 4.670725 | 40 | 0.4 |
| multicellular organismal signaling | 82 | 2.09E-17 | 2.66E-15 | 4.350306 | 42 | 0.42 |
| positive regulation of transport | 86 | 5.04E-16 | 6.12E-14 | 5.371306 | 33 | 0.33 |
| potassium ion export | 87 | 1.31E-15 | 1.57E-13 | 92.97068 | 9 | 0.09 |
| regulation of localization | 88 | 3.28E-15 | 3.89E-13 | 2.885535 | 54 | 0.54 |
| transport | 92 | 4.47E-14 | 5.08E-12 | 2.309987 | 64 | 0.64 |
| cell-cell signaling | 93 | 4.52E-14 | 5.08E-12 | 3.497348 | 42 | 0.42 |
| localization | 96 | 5.50E-14 | 5.98E-12 | 2.029173 | 73 | 0.73 |
| striated muscle tissue development | 97 | 8.86E-14 | 9.54E-12 | 6.773797 | 24 | 0.24 |
| establishment of localization | 99 | 1.25E-13 | 1.32E-11 | 2.263259 | 64 | 0.64 |
| muscle tissue development | 102 | 3.66E-13 | 3.75E-11 | 6.336133 | 24 | 0.24 |
| organ morphogenesis | 103 | 4.42E-13 | 4.48E-11 | 3.526623 | 39 | 0.39 |
| organ development | 104 | 5.23E-13 | 5.25E-11 | 2.230675 | 63 | 0.63 |
| cardiac muscle tissue development | 108 | 5.70E-13 | 5.51E-11 | 8.787839 | 19 | 0.19 |
| relaxation of cardiac muscle | 111 | 1.55E-12 | 1.46E-10 | 41.91772 | 9 | 0.09 |
| positive regulation of potassium ion transmembrane transport | 113 | 3.82E-12 | 3.53E-10 | 85.08963 | 7 | 0.07 |
| regulation of the force of heart contraction | 114 | 4.61E-12 | 4.22E-10 | 27.83993 | 10 | 0.1 |
| heart looping | 115 | 5.15E-12 | 4.68E-10 | 17.85919 | 12 | 0.12 |
| determination of heart left/right asymmetry | 116 | 7.09E-12 | 6.38E-10 | 17.36805 | 12 | 0.12 |
| determination of left/right symmetry | 118 | 1.46E-11 | 1.29E-09 | 12.02453 | 14 | 0.14 |
| His-Purkinje system cell differentiation | 119 | 1.96E-11 | 1.72E-09 | 118.1391 | 6 | 0.06 |
| cardiac pacemaker cell development | 123 | 2.80E-11 | 2.38E-09 | 111.3156 | 6 | 0.06 |
| embryonic heart tube morphogenesis | 124 | 2.98E-11 | 2.51E-09 | 15.31357 | 12 | 0.12 |
| cardiac pacemaker cell differentiation | 128 | 3.72E-11 | 3.04E-09 | 106.1157 | 6 | 0.06 |
| determination of bilateral symmetry | 129 | 4.06E-11 | 3.28E-09 | 11.11383 | 14 | 0.14 |
| relaxation of muscle | 130 | 5.37E-11 | 4.31E-09 | 27.99133 | 9 | 0.09 |
| specification of symmetry | 132 | 6.28E-11 | 4.97E-09 | 10.74598 | 14 | 0.14 |
| regulation of cell proliferation | 133 | 7.05E-11 | 5.53E-09 | 2.804369 | 42 | 0.42 |
| cardiac muscle cell fate commitment | 141 | 3.99E-10 | 2.96E-08 | 71.18162 | 6 | 0.06 |
| embryonic heart tube development | 143 | 6.24E-10 | 4.55E-08 | 11.68972 | 12 | 0.12 |
| regulation of response to stimulus | 144 | 9.02E-10 | 6.54E-08 | 2.076933 | 56 | 0.56 |
| bundle of His development | 149 | 1.61E-09 | 1.13E-07 | 56.23119 | 6 | 0.06 |
| positive regulation of ion transmembrane transport | 152 | 1.69E-09 | 1.16E-07 | 35.14382 | 7 | 0.07 |
| positive regulation of transmembrane transport | 154 | 2.29E-09 | 1.55E-07 | 33.62332 | 7 | 0.07 |
| positive regulation of potassium ion transport | 165 | 1.54E-08 | 9.77E-07 | 25.40195 | 7 | 0.07 |
| regulation of potassium ion transmembrane transport | 169 | 2.68E-08 | 1.66E-06 | 23.41463 | 7 | 0.07 |
| cardiac chamber formation | 170 | 3.47E-08 | 2.13E-06 | 22.53021 | 7 | 0.07 |
| cardiac septum morphogenesis | 180 | 8.38E-08 | 4.86E-06 | 9.898889 | 10 | 0.1 |
| regulation of striated muscle tissue development | 187 | 1.70E-07 | 9.49E-06 | 6.279193 | 13 | 0.13 |
| regulation of muscle organ development | 188 | 1.88E-07 | 1.05E-05 | 6.222137 | 13 | 0.13 |
| cardiac conduction system development | 189 | 1.91E-07 | 1.05E-05 | 24.92734 | 6 | 0.06 |
| regulation of muscle tissue development | 190 | 2.34E-07 | 1.28E-05 | 6.104679 | 13 | 0.13 |
| cardiac chamber morphogenesis | 191 | 2.90E-07 | 1.59E-05 | 6.643675 | 12 | 0.12 |
| striated muscle cell development | 202 | 6.11E-07 | 3.16E-05 | 7.952528 | 10 | 0.1 |
| cardiac muscle cell development | 204 | 7.06E-07 | 3.61E-05 | 11.19376 | 8 | 0.08 |
| atrial septum development | 205 | 7.08E-07 | 3.61E-05 | 19.87219 | 6 | 0.06 |
| cardiac atrium development | 206 | 9.42E-07 | 4.77E-05 | 13.74158 | 7 | 0.07 |
| cardiac cell development | 208 | 1.16E-06 | 5.80E-05 | 10.47255 | 8 | 0.08 |
| negative regulation of gene expression | 210 | 1.22E-06 | 6.06E-05 | 2.507561 | 30 | 0.3 |
| cardiac septum development | 211 | 1.25E-06 | 6.19E-05 | 7.338107 | 10 | 0.1 |
| potassium ion transport | 212 | 1.38E-06 | 6.82E-05 | 5.717796 | 12 | 0.12 |
| muscle cell development | 215 | 1.84E-06 | 8.93E-05 | 7.026002 | 10 | 0.1 |
| negative regulation of multicellular organismal process | 222 | 3.26E-06 | 1.53E-04 | 3.897974 | 16 | 0.16 |
| heart morphogenesis | 223 | 3.33E-06 | 1.56E-04 | 4.436775 | 14 | 0.14 |
| potassium ion transmembrane transport | 224 | 3.33E-06 | 1.55E-04 | 6.566879 | 10 | 0.1 |
| cardiac ventricle morphogenesis | 225 | 3.44E-06 | 1.60E-04 | 7.560801 | 9 | 0.09 |
| tissue development | 229 | 3.55E-06 | 1.62E-04 | 2.127604 | 36 | 0.36 |
| negative regulation of muscle cell differentiation | 231 | 4.34E-06 | 1.96E-04 | 10.87666 | 7 | 0.07 |
| muscle structure development | 234 | 4.71E-06 | 2.10E-04 | 3.262061 | 19 | 0.19 |
| blood vessel development | 239 | 5.84E-06 | 2.55E-04 | 3.08493 | 20 | 0.2 |
| cardiac left ventricle formation | 242 | 6.19E-06 | 2.67E-04 | 88.29244 | 3 | 0.03 |
| venous blood vessel development | 245 | 7.54E-06 | 3.21E-04 | 19.09216 | 5 | 0.05 |
| negative regulation of transcription from RNA polymerase II promoter | 246 | 7.72E-06 | 3.28E-04 | 2.818835 | 22 | 0.22 |
| regulation of transmembrane receptor protein serine/threonine kinase signaling pathway | 253 | 1.03E-05 | 4.25E-04 | 4.687032 | 12 | 0.12 |
| vasculature development | 255 | 1.30E-05 | 5.31E-04 | 2.921622 | 20 | 0.2 |
| striated muscle cell differentiation | 260 | 1.63E-05 | 6.53E-04 | 4.906724 | 11 | 0.11 |
| regulation of cell death | 275 | 3.14E-05 | 1.19E-03 | 2.21892 | 28 | 0.28 |
| negative regulation of macromolecule metabolic process | 277 | 3.36E-05 | 1.27E-03 | 2.018207 | 33 | 0.33 |
| regulation of apoptotic process | 279 | 3.58E-05 | 1.34E-03 | 2.249671 | 27 | 0.27 |
| regulation of programmed cell death | 286 | 4.36E-05 | 1.59E-03 | 2.22473 | 27 | 0.27 |
| regulation of heart growth | 288 | 4.76E-05 | 1.72E-03 | 7.466925 | 7 | 0.07 |
| cardiac muscle tissue morphogenesis | 290 | 5.19E-05 | 1.87E-03 | 7.362849 | 7 | 0.07 |
| cardiac muscle cell differentiation | 294 | 6.12E-05 | 2.17E-03 | 6.01858 | 8 | 0.08 |
| tube morphogenesis | 295 | 6.52E-05 | 2.31E-03 | 3.050725 | 16 | 0.16 |
| cardiac right ventricle morphogenesis | 296 | 6.68E-05 | 2.35E-03 | 12.04926 | 5 | 0.05 |
| adult heart development | 298 | 6.79E-05 | 2.38E-03 | 18.82567 | 4 | 0.04 |
| regulation of organ growth | 300 | 7.41E-05 | 2.58E-03 | 5.854405 | 8 | 0.08 |
| muscle tissue morphogenesis | 303 | 8.02E-05 | 2.76E-03 | 6.865293 | 7 | 0.07 |
| cardiac atrium morphogenesis | 304 | 8.02E-05 | 2.76E-03 | 11.58411 | 5 | 0.05 |
| regulation of intracellular transport | 306 | 8.13E-05 | 2.77E-03 | 3.529166 | 13 | 0.13 |
| ventricular cardiac muscle tissue development | 309 | 8.45E-05 | 2.86E-03 | 8.483715 | 6 | 0.06 |
| morphogenesis of an epithelium | 314 | 9.88E-05 | 3.28E-03 | 2.818979 | 17 | 0.17 |
| regulation of cardiac muscle cell proliferation | 315 | 1.03E-04 | 3.40E-03 | 8.184221 | 6 | 0.06 |
| gap junction assembly | 318 | 1.15E-04 | 3.78E-03 | 32.86493 | 3 | 0.03 |
| muscle organ morphogenesis | 319 | 1.18E-04 | 3.85E-03 | 6.449597 | 7 | 0.07 |
| epithelial tube morphogenesis | 320 | 1.22E-04 | 3.97E-03 | 3.030352 | 15 | 0.15 |
| regulation of cardiac muscle tissue development | 322 | 1.24E-04 | 4.02E-03 | 6.39381 | 7 | 0.07 |
| positive regulation of cardioblast differentiation | 324 | 1.29E-04 | 4.15E-03 | 31.64174 | 3 | 0.03 |
| tissue morphogenesis | 329 | 1.39E-04 | 4.40E-03 | 2.547119 | 19 | 0.19 |
| muscle cell differentiation | 337 | 1.59E-04 | 4.92E-03 | 3.521994 | 12 | 0.12 |
| regulation of cardiac muscle tissue growth | 338 | 1.63E-04 | 5.04E-03 | 7.509512 | 6 | 0.06 |
| negative regulation of nitrogen compound metabolic process | 343 | 1.84E-04 | 5.60E-03 | 2.084846 | 26 | 0.26 |
| negative regulation of transcription, DNA-dependent | 344 | 1.88E-04 | 5.69E-03 | 2.173046 | 24 | 0.24 |
| muscle organ development | 346 | 1.98E-04 | 5.97E-03 | 3.438438 | 12 | 0.12 |
| cardiac ventricle formation | 350 | 2.16E-04 | 6.45E-03 | 13.89182 | 4 | 0.04 |
| positive regulation of stem cell differentiation | 356 | 2.57E-04 | 7.53E-03 | 13.27277 | 4 | 0.04 |
| negative regulation of RNA metabolic process | 357 | 2.71E-04 | 7.92E-03 | 2.120624 | 24 | 0.24 |
| regulation of tight junction assembly | 358 | 2.84E-04 | 8.29E-03 | 24.12491 | 3 | 0.03 |
| cardiocyte differentiation | 366 | 3.53E-04 | 1.01E-02 | 4.645888 | 8 | 0.08 |
| negative regulation of transmembrane receptor protein serine/threonine kinase signaling pathway | 368 | 3.69E-04 | 1.05E-02 | 5.336746 | 7 | 0.07 |
| tube development | 371 | 3.70E-04 | 1.04E-02 | 2.434048 | 18 | 0.18 |
| caveola assembly | 372 | 3.81E-04 | 1.07E-02 | 71.4661 | 2 | 0.02 |
| blood vessel morphogenesis | 373 | 3.88E-04 | 1.09E-02 | 2.719487 | 15 | 0.15 |
| negative regulation of nucleobase-containing compound metabolic process | 374 | 4.08E-04 | 1.14E-02 | 2.021039 | 25 | 0.25 |
| pattern specification process | 385 | 5.79E-04 | 1.57E-02 | 2.513343 | 16 | 0.16 |
| negative regulation of cellular macromolecule biosynthetic process | 387 | 6.09E-04 | 1.64E-02 | 2.00683 | 24 | 0.24 |
| cellular response to extracellular stimulus | 418 | 1.11E-03 | 2.76E-02 | 5.208432 | 6 | 0.06 |
| cellular response to external stimulus | 429 | 1.43E-03 | 3.47E-02 | 4.229112 | 7 | 0.07 |
| lagging strand elongation | 430 | 1.51E-03 | 3.66E-02 | 35.60409 | 2 | 0.02 |

Supplementary Table 5c: GO cellular component

| **# Term Name** | **Binom Rank** | **Binom Raw P-Value** | **Binom FDR Q-Val** | **Binom Fold Enrichment** | **Binom Observed Region Hits** | **Binom Region Set Coverage** |
| --- | --- | --- | --- | --- | --- | --- |
| voltage-gated sodium channel complex | 1 | 2.74E-42 | 3.46E-39 | 206.3409 | 21 | 0.21 |
| sodium channel complex | 2 | 2.60E-41 | 1.64E-38 | 185.28 | 21 | 0.21 |
| cation channel complex | 4 | 7.06E-29 | 2.23E-26 | 15.25014 | 32 | 0.32 |
| caveola | 5 | 1.95E-26 | 4.92E-24 | 27.64923 | 23 | 0.23 |
| ion channel complex | 10 | 2.98E-21 | 3.77E-19 | 8.505308 | 32 | 0.32 |
| integral to plasma membrane | 13 | 7.01E-14 | 6.82E-12 | 3.145442 | 46 | 0.46 |
| intrinsic to plasma membrane | 14 | 2.50E-13 | 2.26E-11 | 3.038717 | 46 | 0.46 |
| protein complex | 19 | 3.65E-12 | 2.43E-10 | 2.114188 | 64 | 0.64 |
| plasma membrane part | 26 | 7.13E-10 | 3.47E-08 | 2.208306 | 52 | 0.52 |
| voltage-gated potassium channel complex | 33 | 3.88E-07 | 1.49E-05 | 8.36341 | 10 | 0.1 |
| basolateral plasma membrane | 42 | 2.18727E-05 | 6.59E-04 | 5.277137 | 10 | 0.1 |

Supplementary Table 5d: Human Phenotype

| **# Term Name** | **Binom Rank** | **Binom Raw P-Value** | **Binom FDR Q-Val** | **Binom Fold Enrichment** | **Binom Observed Region Hits** | **Binom Region Set Coverage** |
| --- | --- | --- | --- | --- | --- | --- |
| Torsade de pointes | 1 | 9.11E-62 | 5.60E-58 | 477.6923 | 26 | 0.26 |
| Ventricular arrhythmia | 7 | 1.27E-55 | 1.11E-52 | 85.1162 | 34 | 0.34 |
| Ventricular fibrillation | 9 | 1.52E-51 | 1.04E-48 | 192.7478 | 26 | 0.26 |
| Prolonged QT interval | 15 | 2.90E-48 | 1.19E-45 | 143.9756 | 26 | 0.26 |
| Atrial fibrillation | 16 | 3.86E-48 | 1.48E-45 | 70.74319 | 31 | 0.31 |
| Primary atrial arrhythmia | 18 | 8.76E-48 | 2.99E-45 | 68.87866 | 31 | 0.31 |
| Supraventricular tachycardia | 19 | 9.02E-48 | 2.92E-45 | 68.81225 | 31 | 0.31 |
| Supraventricular arrhythmia | 20 | 3.04E-47 | 9.34E-45 | 66.14113 | 31 | 0.31 |
| Abnormal EKG | 21 | 3.61E-46 | 1.06E-43 | 88.87661 | 28 | 0.28 |
| Syncope | 22 | 4.92E-46 | 1.38E-43 | 87.89217 | 28 | 0.28 |
| Arrhythmia | 24 | 9.92E-43 | 2.54E-40 | 19.40566 | 42 | 0.42 |
| Palpitations | 26 | 3.21E-42 | 7.59E-40 | 137.1451 | 23 | 0.23 |
| Tachycardia | 27 | 8.96E-42 | 2.04E-39 | 43.83244 | 31 | 0.31 |
| Lipoatrophy | 33 | 1.22E-38 | 2.28E-36 | 53.5989 | 27 | 0.27 |
| Sudden cardiac death | 37 | 1.99E-36 | 3.30E-34 | 44.26903 | 27 | 0.27 |
| Cardiac arrest | 38 | 2.27E-36 | 3.68E-34 | 44.04376 | 27 | 0.27 |
| Abnormality of adipose tissue | 42 | 1.25E-34 | 1.84E-32 | 33.97156 | 28 | 0.28 |
| Abnormality of cardiovascular system physiology | 46 | 8.47E-32 | 1.13E-29 | 9.463796 | 44 | 0.44 |
| Abnormality of cardiac ventricle | 51 | 5.75E-29 | 6.93E-27 | 12.63251 | 35 | 0.35 |
| Autosomal dominant inheritance | 61 | 7.58E-26 | 7.64E-24 | 4.635263 | 56 | 0.56 |
| Malformation of the heart and great vessels | 63 | 7.76E-25 | 7.57E-23 | 6.157488 | 45 | 0.45 |
| Abnormality of the heart | 64 | 1.09E-24 | 1.05E-22 | 6.106177 | 45 | 0.45 |
| Abnormality of the cardiovascular system | 66 | 2.02E-23 | 1.88E-21 | 4.725592 | 51 | 0.51 |
| Abnormality of muscle morphology | 67 | 4.50E-23 | 4.13E-21 | 6.427283 | 41 | 0.41 |
| Abnormality of the vasculature | 75 | 1.38E-19 | 1.13E-17 | 7.459595 | 32 | 0.32 |
| Shortened QT interval | 93 | 2.93E-11 | 1.93E-09 | 63.40217 | 7 | 0.07 |
| Aplasia of the pectoralis major muscle | 103 | 6.97E-09 | 4.16E-07 | 79.66963 | 5 | 0.05 |
| Hypoplastic left heart | 123 | 6.13E-07 | 3.06E-05 | 32.0743 | 5 | 0.05 |
| Hypoplastic heart | 125 | 9.20E-07 | 4.53E-05 | 29.50443 | 5 | 0.05 |
| Abnormality of the aorta | 126 | 1.02E-06 | 4.96E-05 | 7.511795 | 10 | 0.1 |
| Abnormality of the cardiac septa | 132 | 2.11E-06 | 9.82E-05 | 4.617434 | 14 | 0.14 |
| Atrioventricular canal defect | 133 | 2.56E-06 | 1.18E-04 | 23.89849 | 5 | 0.05 |
| Abnormality of the systemic arterial tree | 136 | 3.35E-06 | 1.51E-04 | 5.814202 | 11 | 0.11 |
| Defect in the atrial septum | 173 | 4.10E-05 | 1.46E-03 | 4.896274 | 10 | 0.1 |
| Abnormality of cardiac atrium | 174 | 4.48E-05 | 1.58E-03 | 4.844632 | 10 | 0.1 |
| Skin pits | 269 | 8.28E-04 | 1.89E-03 | 9.696966 | 4 | 0.04 |
| Secundum atrial septal defect | 273 | 9.03E-04 | 2.03E-02 | 46.15891 | 2 | 0.02 |

Supplementary Table 5e: Disease ontology

| **# Term Name** | **Binom Rank** | **Binom Raw P-Value** | **Binom FDR Q-Val** | **Binom Fold Enrichment** | **Binom Observed Region Hits** | **Binom Region Set Coverage** |
| --- | --- | --- | --- | --- | --- | --- |
| sick sinus syndrome | 1 | 3.47E-58 | 7.76E-55 | 1622.77 | 20 | 0.2 |
| sudden infant death syndrome | 3 | 5.18E-54 | 3.86E-51 | 61.69918 | 36 | 0.36 |
| long QT syndrome | 4 | 2.47E-52 | 1.38E-49 | 148.0153 | 28 | 0.28 |
| congestive heart failure | 8 | 5.69E-40 | 1.59E-37 | 47.27614 | 29 | 0.29 |
| complex genetic disease | 9 | 1.32E-36 | 3.27E-34 | 36.02883 | 29 | 0.29 |
| heart failure | 11 | 7.82E-36 | 1.59E-33 | 15.53121 | 39 | 0.39 |
| medical disorder | 13 | 5.40E-28 | 9.28E-26 | 4.972279 | 57 | 0.57 |
| heart disease | 15 | 2.09E-26 | 3.11E-24 | 6.484896 | 46 | 0.46 |
| genetic disease | 23 | 5.60E-18 | 5.44E-16 | 5.538534 | 36 | 0.36 |
| congenital heart defect | 25 | 7.46E-17 | 6.67E-15 | 18.53139 | 17 | 0.17 |
| syndrome | 29 | 5.99E-16 | 4.62E-14 | 2.833575 | 57 | 0.57 |
| Romano-Ward syndrome | 41 | 1.18E-13 | 6.43E-12 | 278.1592 | 6 | 0.06 |
| cardiovascular system disease | 44 | 3.05E-13 | 1.55E-11 | 2.394197 | 59 | 0.59 |
| physical disorder | 54 | 3.28E-10 | 1.36E-08 | 5.322649 | 21 | 0.21 |
| heart septal defect | 65 | 2.28E-08 | 7.84E-07 | 23.97941 | 7 | 0.07 |
| atrial heart septal defect | 74 | 5.11E-07 | 1.54E-05 | 33.29226 | 5 | 0.05 |
| keratosis | 83 | 3.57E-05 | 9.61E-04 | 9.930161 | 6 | 0.06 |
| glaucoma | 88 | 1.93E-04 | 4.92E-03 | 5.94135 | 7 | 0.07 |
| ventricular septal defect | 90 | 2.15E-04 | 05.22E-03 | 26.55344 | 3 | 0.03 |
| nasopharynx carcinoma | 116 | 1.89E-03 | 3.64E-02 | 3.573518 | 8 | 0.08 |
| malignant neoplasm of oropharynx | 119 | 2.24E-03 | 4.22E-02 | 29.01577 | 2 | 0.02 |

**Supplementary Table 6: SNPs that are likely located at a binding site.** Using RegulomeDB, we identified 308 SNPs that likely affect binding because they are located in regulatory regions of the genome. Positions are hg19. The scores reflect likeliness of the SNP to affect binding: 1: likely to affect binding and linked to the expression of a gene target, 2: likely to affect binding, and 3: less likely to affect binding.

Supplementary Table 6a: PR interval

| **Chr** | **Position** | **SNP** | **Score** |
| --- | --- | --- | --- |
| 1 | 112530430 | rs75013985 | 2b |
| 3 | 38172474 | rs116544863 | 2b |
| 3 | 38358796 | rs73064832 | 2b |
| 3 | 38359008 | rs2268750 | 3a |
| 3 | 38561307 | rs17037814 | 2b |
| 3 | 38647047 | rs11711602 | 2b |
| 3 | 38658057 | rs73054554 | 3a |
| 3 | 38685647 | rs7372839 | 2b |
| 3 | 38764782 | rs4076737 | 3a |
| 3 | 38767603 | rs6799257 | 3a |
| 3 | 38771994 | rs9874633 | 2b |
| 3 | 38778191 | rs7428167 | 2b |
| 3 | 38780971 | rs9830687 | 2b |
| 3 | 38796985 | rs12630795 | 2a |
| 3 | 38847152 | rs7627881 | 3a |
| 3 | 39025856 | rs78768764 | 2b |
| 4 | 86623373 | rs1110777 | 2a |
| 4 | 86629102 | rs1871864 | 3a |
| 4 | 86683729 | rs11736641 | 3a |
| 4 | 86699726 | rs13105921 | 3a |
| 5 | 172483023 | rs29775 | 2b |
| 5 | 172502618 | rs2560324 | 2b |
| 7 | 116003990 | rs2157799 | 3a |
| 7 | 116075363 | rs67982517 | 3a |
| 7 | 116078382 | rs34645128 | 2b |
| 7 | 116081121 | rs4730738 | 1f |
| 7 | 116083544 | rs13221364 | 2b |
| 7 | 116088721 | rs1011441 | 3a |
| 7 | 116090300 | rs62471184 | 3a |
| 7 | 116101588 | rs62471189 | 3a |
| 7 | 116104867 | rs35505552 | 3a |
| 7 | 116104911 | rs35210394 | 3a |
| 7 | 116109293 | rs2188243 | 3a |
| 7 | 116117587 | rs35037267 | 3a |
| 7 | 116125834 | rs62468973 | 3a |
| 7 | 116141778 | rs3779511 | 2b |
| 7 | 116142462 | rs71529477 | 2b |
| 7 | 116142808 | rs11980719 | 2b |
| 7 | 116145696 | rs28587043 | 3a |
| 7 | 116145957 | rs4730743 | 3a |
| 7 | 116150077 | rs55701446 | 3a |
| 7 | 116151784 | rs3919515 | 2b |
| 7 | 116169443 | rs7778733 | 3a |
| 7 | 116186241 | rs3807989 | 3a |
| 7 | 116187106 | rs12672038 | 3a |
| 7 | 116187690 | rs12668226 | 3a |
| 7 | 116194905 | rs729949 | 2c |
| 7 | 116203173 | rs10280730 | 3a |
| 7 | 116203323 | rs10232369 | 3a |
| 7 | 116217657 | rs2109517 | 1d |
| 12 | 24753152 | rs1126279 | 2b |
| 12 | 115128655 | rs1910047 | 3a |
| 12 | 115128768 | rs35519587 | 2b |
| 13 | 22115822 | rs11616720 | 3a |
| 13 | 47240876 | rs7986036 | 2b |
| 13 | 47241022 | rs7987387 | 2b |
| 13 | 47241289 | rs7986508 | 3a |
| 13 | 47242088 | rs7993645 | 2b |
| 13 | 47242761 | rs4941564 | 2c |
| 13 | 47243476 | rs1886222 | 3a |
| 14 | 71721797 | rs61989250 | 2c |
| 14 | 71729257 | rs59697713 | 3a |
| 14 | 71788946 | rs12884929 | 2b |
| 14 | 71789862 | rs12891975 | 2b |
| 14 | 71847117 | rs61991243 | 2a |
| 14 | 72022750 | rs2108057 | 2c |
| 14 | 72160321 | rs7148679 | 2b |

Supplementary Table 6b: QRS duration

| **Chr** | **Position** | **SNP** | **Score** |
| --- | --- | --- | --- |
| 1 | 61909126 | rs12741766 | 3a |
| 3 | 38172474 | rs116544863 | 2b |
| 3 | 38647047 | rs11711602 | 2b |
| 3 | 38658057 | rs73054554 | 3a |
| 3 | 38685647 | rs7372839 | 2b |
| 3 | 38764782 | rs4076737 | 3a |
| 3 | 38778191 | rs7428167 | 2b |
| 5 | 153871832 | rs10054375 | 2c |
| 5 | 153871841 | rs10076436 | 2c |
| 6 | 36617652 | rs12207916 | 2b |
| 6 | 36618821 | rs1321313 | 2b |
| 6 | 36621533 | rs4713994 | 1f |
| 6 | 36622900 | rs1321311 | 1f |
| 6 | 36623124 | rs1321310 | 1d |
| 6 | 36625272 | rs6930671 | 1f |
| 6 | 36625382 | rs11969445 | 1d |
| 6 | 36626322 | rs6936993 | 1f |
| 6 | 36628953 | rs9462210 | 3a |
| 6 | 36629444 | rs10807170 | 1f |
| 6 | 36629714 | rs4713996 | 1f |
| 6 | 36630525 | rs9394368 | 1f |
| 6 | 36632688 | rs13196885 | 2b |
| 6 | 36634156 | rs6930083 | 1f |
| 6 | 36636080 | rs66761782 | 2b |
| 6 | 36638175 | rs4714001 | 1f |
| 6 | 36638636 | rs1321309 | 1f |
| 6 | 36638691 | rs1321308 | 1f |
| 6 | 36645203 | rs733590 | 1f |
| 6 | 36645696 | rs2395655 | 1f |
| 6 | 36647680 | rs4135240 | 2b |
| 6 | 36648920 | rs3176337 | 2b |
| 6 | 36656256 | rs12207548 | 3a |
| 6 | 36668768 | rs12528085 | 3a |
| 6 | 36695519 | rs236472 | 3a |
| 6 | 36695661 | rs236471 | 1f |
| 6 | 36696330 | rs236470 | 3b |
| 6 | 36697201 | rs236467 | 1b |
| 6 | 36700437 | rs86702 | 1f |
| 6 | 118570990 | rs281872 | 3a |
| 6 | 118606000 | rs283080 | 2b |
| 6 | 118827252 | rs62424199 | 3a |
| 6 | 118846022 | rs9481821 | 3a |
| 6 | 118863789 | rs12206329 | 3a |
| 6 | 118876092 | rs9481825 | 1f |
| 6 | 118884092 | rs12197337 | 3a |
| 6 | 118884098 | rs541442 | 3a |
| 6 | 118895481 | rs72952798 | 3a |
| 6 | 118971913 | rs62422235 | 2b |
| 6 | 118986309 | rs9320665 | 3a |
| 6 | 118998481 | rs2638550 | 3a |
| 6 | 119027325 | rs7746210 | 2b |
| 7 | 35302017 | rs1362209 | 2b |
| 10 | 114455827 | rs6585173 | 1f |
| 10 | 114468438 | rs7907540 | 1f |
| 10 | 114481022 | rs17585548 | 2b |
| 10 | 114482409 | rs6585179 | 3a |
| 10 | 114506292 | rs7096151 | 1f |
| 10 | 114514051 | rs6585184 | 3a |
| 14 | 71698616 | rs7156194 | 1b |
| 14 | 71704261 | rs57204407 | 2b |
| 14 | 71704684 | rs7160587 | 2b |
| 14 | 71705928 | rs61989239 | 3a |
| 14 | 71712185 | rs55896578 | 3a |
| 14 | 71721797 | rs61989250 | 2c |
| 14 | 71729257 | rs59697713 | 3a |
| 14 | 71733790 | rs17767362 | 1f |
| 14 | 71757679 | rs68073803 | 2b |
| 14 | 71767848 | rs71305837 | 3a |
| 14 | 71788946 | rs12884929 | 2b |
| 14 | 71789862 | rs12891975 | 2b |
| 14 | 71847117 | rs61991243 | 2a |
| 14 | 71861278 | rs34528131 | 3a |
| 14 | 72022750 | rs2108057 | 2c |
| 14 | 72023489 | rs7142343 | 3a |
| 14 | 72030809 | rs10135680 | 3a |
| 14 | 72160321 | rs7148679 | 2b |

Supplementary Table 6c: QT interval

| **Chr** | **Position** | **SNP** | **Score** |
| --- | --- | --- | --- |
| 1 | 162005477 | rs75192393 | 2b |
| 1 | 162006862 | rs12123710 | 3a |
| 1 | 162010693 | rs6666546 | 2b |
| 1 | 162010787 | rs10158975 | 3a |
| 1 | 162024987 | rs10429888 | 2b |
| 1 | 162027992 | rs7547308 | 3a |
| 1 | 162033890 | rs12143842 | 2b |
| 1 | 162040305 | rs12096347 | 2b |
| 1 | 162040878 | rs16849113 | 3a |
| 1 | 162050670 | rs144846894 | 3a |
| 1 | 162101829 | rs12093845 | 1f |
| 1 | 162103510 | rs10918723 | 3a |
| 1 | 162103672 | rs10918724 | 3a |
| 1 | 162103772 | rs61007280 | 3a |
| 1 | 162104658 | rs1337072 | 1d |
| 1 | 162104763 | rs1337071 | 1f |
| 1 | 162105094 | rs6663393 | 1f |
| 1 | 162106150 | rs10918732 | 1f |
| 1 | 162106525 | rs12090201 | 1f |
| 1 | 162106636 | rs12087337 | 1f |
| 1 | 162106962 | rs1415265 | 2b |
| 1 | 162106967 | rs1415264 | 2b |
| 1 | 162112082 | rs59127492 | 3a |
| 1 | 162118984 | rs10918762 | 3a |
| 1 | 162122448 | rs12064771 | 1a |
| 1 | 162129466 | rs144958472 | 2b |
| 1 | 162129546 | rs12068421 | 2b |
| 1 | 162166919 | rs6663969 | 2b |
| 1 | 162182677 | rs3934467 | 3a |
| 1 | 162185546 | rs4657173 | 2b |
| 1 | 162191412 | rs6683968 | 1f |
| 1 | 162194498 | rs3923374 | 1f |
| 1 | 162196530 | rs3923368 | 1f |
| 1 | 162196574 | rs3923367 | 1f |
| 1 | 162210610 | rs4657178 | 3a |
| 1 | 162217432 | rs7541606 | 2b |
| 1 | 162217459 | rs10918974 | 3a |
| 1 | 162219101 | rs4656362 | 2b |
| 1 | 162249697 | rs6659953 | 3a |
| 1 | 162249715 | rs6670958 | 2c |
| 1 | 162255112 | rs10800404 | 2b |
| 1 | 162255286 | rs6680461 | 3a |
| 1 | 162255385 | rs4657181 | 2b |
| 1 | 162263270 | rs10919096 | 2b |
| 1 | 162263712 | rs6692467 | 3a |
| 1 | 162263714 | rs386636157 | 3a |
| 1 | 162317513 | rs347273 | 2b |
| 1 | 162319524 | rs11577628 | 3a |
| 1 | 169072992 | rs79011457 | 2b |
| 1 | 169073002 | rs12079856 | 2b |
| 1 | 169073384 | rs1892093 | 3a |
| 1 | 169073388 | rs1320977 | 3a |
| 1 | 169074268 | rs75505858 | 2b |
| 1 | 169074736 | rs1534984 | 2b |
| 1 | 169075348 | rs12751593 | 2b |
| 1 | 169079020 | rs1200133 | 2b |
| 1 | 169088679 | rs2143290 | 1f |
| 1 | 169088947 | rs10919062 | 1f |
| 1 | 169098734 | rs72706963 | 3a |
| 1 | 169099037 | rs10919070 | 1f |
| 1 | 169102340 | rs12035622 | 1f |
| 1 | 169163017 | rs3766074 | 2b |
| 1 | 169219185 | rs114089179 | 3a |
| 1 | 169453703 | rs72706084 | 2b |
| 1 | 169455435 | rs2056926 | 2b |
| 6 | 118570990 | rs281872 | 3a |
| 6 | 118606000 | rs283080 | 2b |
| 6 | 118630300 | rs283043 | 3a |
| 6 | 118827252 | rs62424199 | 3a |
| 6 | 118846022 | rs9481821 | 3a |
| 6 | 118847551 | rs79477297 | 3a |
| 6 | 118863789 | rs12206329 | 3a |
| 6 | 118876092 | rs9481825 | 1f |
| 6 | 118884092 | rs12197337 | 3a |
| 6 | 118884098 | rs541442 | 3a |
| 6 | 118895481 | rs72952798 | 3a |
| 6 | 118901793 | rs9489448 | 2c |
| 6 | 118971913 | rs62422235 | 2b |
| 6 | 118973953 | rs9489486 | 3a |
| 6 | 118986309 | rs9320665 | 3a |
| 6 | 118998481 | rs2638550 | 3a |
| 6 | 118998633 | rs17349133 | 3a |
| 6 | 119007427 | rs62422258 | 3a |
| 6 | 119027325 | rs7746210 | 2b |
| 7 | 150573236 | rs4725974 | 3a |
| 7 | 150573270 | rs35399955 | 3a |
| 7 | 150620701 | rs6972137 | 2b |
| 7 | 150640285 | rs1547958 | 2b |
| 7 | 150644394 | rs3815459 | 2b |
| 7 | 150655643 | rs758890 | 2b |
| 7 | 150657201 | rs12668582 | 3a |
| 7 | 150657209 | rs6947240 | 1f |
| 7 | 150658678 | rs35760656 | 3a |
| 7 | 150659051 | rs3778874 | 2b |
| 7 | 150661633 | rs7789585 | 3a |
| 11 | 2484803 | rs2074238 | 2a |
| 11 | 61549458 | rs174534 | 1b |
| 11 | 61565908 | rs174541 | 3a |
| 11 | 61569830 | rs174546 | 1f |
| 11 | 61570783 | rs174547 | 1d |
| 11 | 61571348 | rs174548 | 1f |
| 11 | 61571382 | rs174549 | 1f |
| 11 | 61579463 | rs174554 | 3a |
| 11 | 61579760 | rs174555 | 1f |
| 11 | 61580635 | rs174556 | 1f |
| 11 | 61582708 | rs174561 | 2b |
| 11 | 61593816 | rs174568 | 1f |
| 11 | 61596633 | rs99780 | 2b |
| 11 | 61597972 | rs1535 | 1b |
| 11 | 61603510 | rs174576 | 1f |
| 11 | 61604814 | rs174577 | 1f |
| 11 | 61609750 | rs174583 | 1f |
| 16 | 11692658 | rs7191330 | 1f |
| 16 | 11701021 | rs72781039 | 3a |
| 16 | 11707291 | rs9932684 | 1a |
| 16 | 11707567 | rs9932278 | 2b |
| 16 | 58529615 | rs4356470 | 1f |
| 16 | 58549932 | rs4784046 | 2b |
| 16 | 58550052 | rs185639574 | 2b |
| 16 | 58616984 | rs17854029 | 3a |
| 16 | 58648453 | rs28580327 | 3a |
| 16 | 58649139 | rs1549605 | 3a |
| 16 | 58671928 | rs149166 | 3a |
| 16 | 58686600 | rs154439 | 2b |
| 17 | 33307586 | rs12945428 | 2b |
| 17 | 33324382 | rs2074518 | 1f |
| 17 | 33331575 | rs1052536 | 1f |
| 17 | 33332629 | rs12948362 | 1f |
| 17 | 33341834 | rs2074519 | 1f |
| 17 | 33353332 | rs3926358 | 1f |
| 17 | 33382801 | rs2339122 | 1f |
| 17 | 33383030 | rs1634800 | 1f |
| 17 | 33414758 | rs797989 | 1f |
| 21 | 35969194 | rs112728994 | 3a |

Supplementary Table 6d: RR interval

| **Chr** | **Position** | **SNP** | **Score** |
| --- | --- | --- | --- |
| 1 | 208136236 | rs650470 | 2b |
| 6 | 121837314 | rs9385199 | 3a |
| 6 | 121941164 | rs55993325 | 3a |
| 6 | 121984911 | rs11755453 | 3a |
| 6 | 122138479 | rs9388008 | 2b |
| 6 | 122149831 | rs9375064 | 3a |
| 6 | 122171052 | rs1919875 | 2b |
| 6 | 122179724 | rs79047865 | 3a |
| 7 | 100465355 | rs12705089 | 2b |
| 7 | 100473550 | rs12705092 | 2b |
| 7 | 100494949 | rs17883557 | 2b |
| 7 | 100494959 | rs17881088 | 2b |
| 7 | 100494960 | rs76181418 | 2b |
| 7 | 100516003 | rs10278546 | 1f |
| 7 | 100518458 | rs12705099 | 1f |
| 12 | 33516963 | rs1905408 | 3a |
| 12 | 33529288 | rs9888363 | 3b |
| 12 | 33593127 | rs6488162 | 3a |
| 14 | 23868285 | rs439735 | 1f |
| 14 | 23873092 | rs388914 | 1f |
| 14 | 23874523 | rs2277474 | 1f |
| 14 | 23882144 | rs2284651 | 1f |
| 14 | 23882855 | rs2331979 | 1f |
| 14 | 23885887 | rs3729829 | 1f |
| 14 | 23888040 | rs12894524 | 1f |
| 14 | 23890982 | rs743567 | 1f |
| 20 | 36838422 | rs6127448 | 2b |
| 20 | 36839297 | rs3746467 | 2b |
| 20 | 36841914 | rs3746471 | 3a |
| 20 | 36842836 | rs6024001 | 3a |
| 20 | 36843334 | rs6127467 | 3a |

**References**

1. Pardo LM, MacKay I, Oostra B, van Duijn CM, Aulchenko YS. The effect of genetic drift in a young genetically isolated population. *Ann Hum Genet*. 2005;69:288-295.

2. Scholtens S, Smidt N, Swertz MA, Bakker SJ, Dotinga A, Vonk JM, et al. Cohort profile: Lifelines, a three-generation cohort study and biobank. *Int J Epidemiol*. 2015;44:1172-1180.

3. Boomsma DI, de Geus EJ, Vink JM, Stubbe JH, Distel MA, Hottenga JJ, et al. Netherlands twin register: From twins to twin families. *Twin Res Hum Genet*. 2006;9:849-857.

4. Willemsen G, de Geus EJ, Bartels M, van Beijsterveldt CE, Brooks AI, Estourgie-van Burk GF, et al. The netherlands twin register biobank: A resource for genetic epidemiological studies. *Twin Res Hum Genet*. 2010;13:231-245.

5. Hillege HL, Fidler V, Diercks GF, van Gilst WH, de Zeeuw D, van Veldhuisen DJ, et al. Urinary albumin excretion predicts cardiovascular and noncardiovascular mortality in general population. *Circulation*. 2002;106:1777-1782.

6. Shepherd J, Blauw GJ, Murphy MB, Cobbe SM, Bollen EL, Buckley BM, et al. The design of a prospective study of pravastatin in the elderly at risk (prosper). Prosper study group. Prospective study of pravastatin in the elderly at risk. *Am J Cardiol*. 1999;84:1192-1197.

7. Shepherd J, Blauw GJ, Murphy MB, Bollen EL, Buckley BM, Cobbe SM, et al. Pravastatin in elderly individuals at risk of vascular disease (prosper): A randomised controlled trial. *Lancet*. 2002;360:1623-1630.

8. Trompet S, de Craen AJ, Postmus I, Ford I, Sattar N, Caslake M, et al. Replication of ldl gwas hits in prosper/phase as validation for future (pharmaco)genetic analyses. *BMC Med Genet*. 2011;12:131.

9. Ikram MA, van der Lugt A, Niessen WJ, Krestin GP, Koudstaal PJ, Hofman A, et al. The rotterdam scan study: Design and update up to 2012. *Eur J Epidemiol*. 2011;26:811-824.

10. Harris TB, Launer LJ, Eiriksdottir G, Kjartansson O, Jonsson PV, Sigurdsson G, et al. Age, gene/environment susceptibility-reykjavik study: Multidisciplinary applied phenomics. *Am J Epidemiol*. 2007;165:1076-1087.

11. The atherosclerosis risk in communities (aric) study: Design and objectives. The aric investigators. *Am J Epidemiol*. 1989;129:687-702.

12. Caulfield M, Munroe P, Pembroke J, Samani N, Dominiczak A, Brown M, et al. Genome-wide mapping of human loci for essential hypertension. *Lancet*. 2003;361:2118-2123.

13. Fried LP, Borhani NO, Enright P, Furberg CD, Gardin JM, Kronmal RA, et al. The cardiovascular health study: Design and rationale. *Ann Epidemiol*. 1991;1:263-276.

14. Tobin MD, Tomaszewski M, Braund PS, Hajat C, Raleigh SM, Palmer TM, et al. Common variants in genes underlying monogenic hypertension and hypotension and blood pressure in the general population. *Hypertension*. 2008;51:1658-1664.

15. Girotto G, Pirastu N, Sorice R, Biino G, Campbell H, d'Adamo AP, et al. Hearing function and thresholds: A genome-wide association study in european isolated populations identifies new loci and pathways. *J Med Genet*. 2011;48:369-374.

16. Jorgensen T, Borch-Johnsen K, Thomsen TF, Ibsen H, Glumer C, Pisinger C. A randomized non-pharmacological intervention study for prevention of ischaemic heart disease: Baseline results inter99. *Eur J Cardiovasc Prev Rehabil*. 2003;10:377-386.

17. Taylor HA, Jr., Wilson JG, Jones DW, Sarpong DF, Srinivasan A, Garrison RJ, et al. Toward resolution of cardiovascular health disparities in african americans: Design and methods of the jackson heart study. *Ethn Dis*. 2005;15:S6-4-17.

18. Holle R, Happich M, Lowel H, Wichmann HE, Group MKS. Kora--a research platform for population based health research. *Gesundheitswesen*. 2005;67 Suppl 1:S19-25.

19. Wichmann HE, Gieger C, Illig T, Group MKS. Kora-gen--resource for population genetics, controls and a broad spectrum of disease phenotypes. *Gesundheitswesen*. 2005;67 Suppl 1:S26-30.

20. Moayyeri A, Hammond CJ, Hart DJ, Spector TD. The uk adult twin registry (twinsuk resource). *Twin Res Hum Genet*. 2013;16:144-149.

21. Raitakari OT, Juonala M, Ronnemaa T, Keltikangas-Jarvinen L, Rasanen L, Pietikainen M, et al. Cohort profile: The cardiovascular risk in young finns study. *Int J Epidemiol*. 2008;37:1220-1226.

22. McLean CY, Bristor D, Hiller M, Clarke SL, Schaar BT, Lowe CB, et al. Great improves functional interpretation of cis-regulatory regions. *Nat Biotechnol*. 2010;28:495-501.

**Funding**

The Age, Gene/Environment Susceptibility Reykjavik Study is funded by National Institutes of Health [N01-AG-12100], the NIA Intramural Research Program, Hjartavernd (the Icelandic Heart Association), and the Althingi (the Icelandic Parliament), in addition an Intramural Research Program Award [ZIAEY000401] from the National Eye Institute, an award from the National Institute on Deafness and Other Communication Disorders (NIDCD) Division of Scientific Programs [IAA Y2-DC_1004-02]. The study is approved by the Icelandic National Bioethics Committee, VSN: 00-063.

The Atherosclerosis Risk in Communities Study is carried out as a collaborative study supported by National Heart, Lung, and Blood Institute contracts [HHSN268201100005C, HHSN268201100006C, HHSN268201100007C, HHSN268201100008C, HHSN268201100009C, HHSN268201100010C, HHSN268201100011C, and HHSN268201100012C, R01HL087641, R01HL59367 and R01HL086694]; National Human Genome Research Institute [contract U01HG004402]; and National Institutes of Health ;contract HHSN268200625226C]. Infrastructure was partly supported by Grant Number UL1RR025005, a component of the National Institutes of Health and NIH Roadmap for Medical Research. Additional funding was provided by American Heart Association grant [16EIA26410001] (A.A.).

BRIGHT was funded by the Medical Research Council of Great Britain [grant number: G9521010D]. This work forms part of the research themes contributing to the translational research portfolio for the NIHR Barts Cardiovascular Biomedical Research Unit. The funders had no role in study design, data collection and analysis.

This CHS research was supported by National Heart, Lung, and Blood Institute [contracts HHSN268201200036C, HHSN268200800007C, N01HC55222, N01HC85079, N01HC85080, N01HC85081, N01HC85082, N01HC85083, N01HC85086, HHSN268200960009C, HL080295, HL087652, HL105756, HL103612] with additional contribution from the National Institute of Neurological Disorders and Stroke (NINDS). Additional support was provided through AG023629 from the National Institute on Aging (NIA). A full list of principal CHS investigators and institutions can be found at CHS-NHLBI.org/.

The ERF study as a part of EUROSPAN (European Special Populations Research Network) was supported by European Commission FP6 STRP grant number 018947 [LSHG-CT-2006-01947] and also received funding from the European Community's Seventh Framework Programme (FP7/2007-2013)/grant agreement HEALTH-F4-2007-201413 by the European Commission under the programme “Quality of Life and Management of the Living Resources” of 5th Framework Programme [no. QLG2-CT-2002-01254]. The ERF study was further supported by ENGAGE consortium and CMSB. High-throughput analysis of the ERF data was supported by joint grant from Netherlands Organisation for Scientific Research and the Russian Foundation for Basic Research [NWO-RFBR 047.017.043].

The GRAPHIC study was funded by the British Heart Foundation.

Inter99 was financially supported by research grants from the Danish Research Council, the Danish Centre for Health Technology Assessment, Novo Nordisk Inc., Research Foundation of Copenhagen County, Ministry of Internal Affairs and Health, the Danish Heart Foundation, the Danish Pharmaceutical Association, the Augustinus Foundation, the Ib Henriksen Foundation, the Becket Foundation, and the Danish Diabetes Association. The Novo Nordisk Foundation Center for Basic Metabolic Research is an independent Research Center at the University of Copenhagen partially funded by an unrestricted donation from the Novo Nordisk Foundation ([www.metabol.ku.dk](http://www.metabol.ku.dk)).

The JHS is supported by the National Heart, Lung, and Blood Institute [contracts HHSN268201300046C, HHSN268201300047C, HHSN268201300048C, HHSN268201300049C, HHSN268201300050C] and the National Institute on Minority Health and Health Disparities. J.G.W. is supported by the National Institute of General Medical Sciences [U54GM115428].

The LifeLines Cohort Study, and generation and management of GWAS genotype data for the LifeLines Cohort Study is supported by the Netherlands Organization of Scientific Research NWO [grant 175.010.2007.006], the Economic Structure Enhancing Fund (FES) of the Dutch government, the Ministry of Economic Affairs, the Ministry of Education, Culture and Science, the Ministry for Health, Welfare and Sports, the Northern Netherlands Collaboration of Provinces (SNN), the Province of Groningen, University Medical Center Groningen, the University of Groningen, Dutch Kidney Foundation and Dutch Diabetes Research Foundation. N.V. is supported by Marie Sklodowska-Curie GF [call: H2020-MSCA-IF-2014, Project ID: 661395]

The KORA study was initiated and financed by the Helmholtz Zentrum München – German Research Center for Environmental Health, which is funded by the German Federal Ministry of Education and Research (BMBF) and by the State of Bavaria. Furthermore, KORA research was supported within the Munich Center of Health Sciences (MC-Health), Ludwig-Maximilians-Universität, as part of LMUinnovativ.

Netherland Twin Register (NTR): Funding was obtained from the Netherlands Organization for Scientific Research (NWO) and The Netherlands Organisation for Health Research and Development (ZonMW) grants 904-61-090, 985-10-002, 912-10-020, 904-61-193,480-04-004,  463-06-001, 451-04-034, 400-05-717,  Addiction-31160008, 016-115-035, 481-08-011, 056-32-010, Middelgroot-911-09-032,  OCW_NWO Gravity program –024.001.003, NWO-Groot 480-15-001/674,  Center for Medical Systems Biology (CSMB, NWO Genomics), NBIC/BioAssist/RK(2008.024), Biobanking and Biomolecular Resources Research Infrastructure (BBMRI –NL, 184.021.007 an 184.033.111);  Spinozapremie (NWO- 56-464-14192),  KNAW Academy Professor Award (PAH/6635) and University Research Fellow grant (URF) to DIB; Amsterdam Public Health research institute (former EMGO+) , Neuroscience Amsterdam research institute (former NCA) ; the European Science Foundation (ESF, EU/QLRT-2001-01254), the European Community's Seventh Framework Program (FP7- HEALTH-F4-2007-2013, grant 01413: ENGAGE and grant 602768: ACTION);  the European Research Council (ERC Advanced, 230374, ERC Starting grant 284167), Rutgers University Cell and DNA Repository (NIMH U24 MH068457-06),  the National Institutes of Health (NIH, R01D0042157-01A1, R01MH58799-03, MH081802,  DA018673, R01 DK092127-04, Grand Opportunity grants 1RC2 MH089951, and 1RC2 MH089995);  the Avera Institute for Human Genetics, Sioux Falls, South Dakota (USA). Part of the genotyping and analyses were funded by the Genetic Association Information Network (GAIN) of the Foundation for the National Institutes of Health. Computing was supported by NWO through grant 2018/EW/00408559, BiG Grid, the Dutch e-Science Grid and SURFSARA.

PREVEND genetics is supported by the Dutch Kidney Foundation [Grant E033], the National Institutes of Health [grant LM010098], The Netherlands Organization for Scientific Research [NWO-Groot 175.010.2007.006, NWO VENI grant 916.761.70 and NWO VIDI grant 917.13.350], ZonMW [90.700.441], and the Dutch Inter University Cardiology Institute Netherlands.

The PROSPER study was supported by an investigator initiated grant obtained from Bristol-Myers Squibb. Prof. Dr. J. W. Jukema is an Established Clinical Investigator of the Netherlands Heart Foundation [grant 2001 D 032]. Support for genotyping was provided by the seventh framework program of the European commission [grant 223004] and by the Netherlands Genomics Initiative [Netherlands Consortium for Healthy Aging grant 050-060-810].

The generation and management of GWAS genotype data for the Rotterdam Study (RS I, RS II, RS III) was executed by the Human Genotyping Facility of the Genetic Laboratory of the Department of Internal Medicine, Erasmus MC, Rotterdam, The Netherlands. The GWAS datasets are supported by the Netherlands Organisation of Scientific Research NWO Investments [nr. 175.010.2005.011, 911-03-012], the Genetic Laboratory of the Department of Internal Medicine, Erasmus MC, the Research Institute for Diseases in the Elderly [014-93-015; RIDE2], the Netherlands Genomics Initiative (NGI)/Netherlands Organisation for Scientific Research (NWO) Netherlands Consortium for Healthy Aging (NCHA) [project nr. 050-060-810]. The Rotterdam Study is funded by Erasmus Medical Center and Erasmus University, Rotterdam, Netherlands Organization for the Health Research and Development (ZonMw), the Research Institute for Diseases in the Elderly (RIDE), the Ministry of Education, Culture and Science, the Ministry for Health, Welfare and Sports, the European Commission (DG XII), and the Municipality of Rotterdam.

TwinsUK was funded by a grant from the British Heart Foundation [PG/12/38/29615].

The Young Finns Study has been financially supported by the Academy of Finland [grants 286284, 134309 (Eye), 126925, 121584, 124282, 129378 (Salve), 117787 (Gendi), and 41071 (Skidi)]; the Social Insurance Institution of Finland; Kuopio, Tampere and Turku University Hospital Medical Funds [grant X51001]; Juho Vainio Foundation; Paavo Nurmi Foundation; Finnish Foundation of Cardiovascular Research; Finnish Cultural Foundation; Tampere Tuberculosis Foundation; Emil Aaltonen Foundation; and Yrjö Jahnsson Foundation.

**Acknowledgments**

**AGES**

The researchers are indebted to the participants for their willingness to participate in the study.

**ARIC**

The authors thank the staff and participants of the ARIC study for their important contributions.

**BRIGHT**

The BRIGHT study is extremely grateful to all the patients who participated in the study and the BRIGHT nursing team.

**ERF**

We are grateful to all study participants and their relatives, general practitioners and neurologists for their contributions to the ERF study and to P Veraart for her help in genealogy, J Vergeer for the supervision of the laboratory work and P Snijders for his help in data collection.

**Inter99**

The Inter99 was initiated by Torben Jørgensen (PI), Knut Borch-Johnsen (co-PI), Hans Ibsen and Troels F. Thomsen. The steering committee comprises the former two and Charlotta Pisinger.

**JHS**

We thank the Jackson Heart Study (JHS) participants and staff for their contributions to this work.

**RS**

We thank Pascal Arp, Mila Jhamai, Marijn Verkerk, Lizbeth Herrera and Marjolein Peters, MSc, and Carolina Medina-Gomez, MSc, for their help in creating the GWAS database, and Karol Estrada, PhD, Yurii Aulchenko, PhD, and Carolina Medina-Gomez, MSc, for the creation and analysis of imputed data. The authors are grateful to the study participants, the staff from the Rotterdam Study and the participating general practitioners and pharmacists. We would like to thank Karol Estrada PhD, Fernando Rivadeneira PhD, and Anis Abuseiris (Erasmus MC Rotterdam, The Netherlands), for their help in creating GRIMP, and we thank BigGRID for access to their grid computing resources.

**YFS**

The expert technical assistance in the statistical analyses by Irina Lisinen is gratefully acknowledged.
